# Modeling Early Phases of COVID-19 Pandemic in Northern Italy and Its Implication for Outbreak Diffusion

**DOI:** 10.3389/fpubh.2021.724362

**Published:** 2021-12-16

**Authors:** Daniela Gandolfi, Giuseppe Pagnoni, Tommaso Filippini, Alessia Goffi, Marco Vinceti, Egidio D'Angelo, Jonathan Mapelli

**Affiliations:** ^1^Department of Biomedical, Metabolic and Neural Sciences, University of Modena and Reggio Emilia, Modena, Italy; ^2^Center for Neuroscience and Neurotechnology, University of Modena and Reggio Emilia, Modena, Italy; ^3^TerrAria, Milan, Italy; ^4^Department of Epidemiology, Boston University School of Public Health, Boston, MA, United States; ^5^Department of Brain and Behavioral Sciences, University of Pavia, Pavia, Italy; ^6^Brain Connectivity Center, Istituto di Ricovero e Cura a Carattere Scientifico (IRCCS) Mondino Foundation, Pavia, Italy

**Keywords:** DCM—dynamic causal modeling, COVID-19, predictive modeling, computational modeling, brain modeling

## Abstract

The COVID-19 pandemic has sparked an intense debate about the hidden factors underlying the dynamics of the outbreak. Several computational models have been proposed to inform effective social and healthcare strategies. Crucially, the predictive validity of these models often depends upon incorporating behavioral and social responses to infection. Among these tools, the analytic framework known as “dynamic causal modeling” (DCM) has been applied to the COVID-19 pandemic, shedding new light on the factors underlying the dynamics of the outbreak. We have applied DCM to data from northern Italian regions, the first areas in Europe to contend with the outbreak, and analyzed the predictive validity of the model and also its suitability in highlighting the hidden factors governing the pandemic diffusion. By taking into account data from the beginning of the pandemic, the model could faithfully predict the dynamics of outbreak diffusion varying from region to region. The DCM appears to be a reliable tool to investigate the mechanisms governing the spread of the SARS-CoV-2 to identify the containment and control strategies that could efficiently be used to counteract further waves of infection.

## Introduction

The COVID-19 pandemic has engendered a key debate about the generative factors governing its evolution, and a wide series of mathematical models have been developed to predict the evolution of the outbreak. The fight against the virus has in fact benefited from reliable models explaining the wealth of epidemiological data to the aim of informing the healthcare system. Italy was the first country severely hit by the outbreak outside China, particularly in its northern part, and has promptly adopted a tight lockdown strategy and other specific public health measures (1, 2). Nonetheless, northern Italian regions were more affected than others, with variable severity, raising questions that remain so far unanswered: (i) How did the tight lockdown impact the local dynamics of SARS-CoV-2 spread? (ii) Why were there substantial differences across regions despite the similar public health measures adopted? and (iii) Could have been possible to predict somehow the incoming of subsequent waves?

Relevant epidemiological data have been accumulated during the pandemic diffusion (3–5), and predictive models have been developed to inform social and healthcare strategies. Compartmental models such as the SIR-based (susceptible–infected–recovered) or SEIR-based (susceptible–exposed–infectious–recovered) models have been used during the SARS-CoV-2 outbreak (6–12)–which are generally based on differential equations accounting for the rate of transition of an individual between specific states or compartments. The extension of SIR models with network linkages, in which the statistical dependencies between contacts are part of model structure, demonstrates a notable improvement in the estimation of transmission parameters (12). Recently, the application of the dynamic causal modeling (DCM) (13) to the COVID-19 pandemic allowed to extract different interpretative perspectives on the factors driving the pandemic through different countries and phases (14–17). DCM is a flexible statistical procedure, originally designed to infer the nature of connectivity in brain networks. Whereas, DCM originates in the neuroimaging field to infer the nature of connectivity in brain networks, it is grounded on a generic theoretical–computational framework, which can be applied to a variety of non-linear dynamical systems where different causal sources interact in complex ways. In detail, DCM involves positing an architecture of coupled causes that interact in generating observable quantities. The causes themselves are not directly observable (they are “latent”) but are probabilistically inferred by the model so as to explain the observable data in a Bayes-optimal fashion. This methodology allows one to model the temporal trajectory of several quantities related to the process under study and to estimate the impact of the underlying latent factors on observable data. In DCM, compartmental models are implemented as a generative model, where transitions among compartments are equivalent to the pathways of information transferred between the latent factors. DCM, therefore, can be aptly used to study the behavior of a network of compartments, allowing their dynamical interaction, along with the estimation of a large set of hidden parameters, under standard frameworks of Bayesian inference and parameter optimization.

The DCM has been applied to the evolution of the outbreaks in different countries on a worldwide scale, demonstrating remarkable performance in terms of goodness of fit and predictive validity and allowing to predict the upsurge of cases in several European countries and also the dynamics of the second waves (17). In this study, we have adopted a similar procedure on a finer-grain scale, by adapting it to northern Italian regions which are characterized by a high degree of heterogeneity (18). A DCM analysis of the COVID-19 pandemic is of special interest since, in addition to characterizing the latent causes of regional differences, has the potential to uncover the dependencies between the efficacy of testing and tracking strategy and the second-wave dynamics.

This study addresses the following aims: (1) validating the DCM in modeling the spread of COVID-19 in northern Italy, (2) inferring the relative weight of the latent causal factors influencing the evolution of the pandemic in northern Italian regions, (3) applying the DCM as a reliable predictive tool to evaluate a strategic intervention to suggest the implementation of public health measures.

## Results

### Estimation of the Dynamics of Pandemic's Waves

One of the main unknowns regarding the COVID-19 pandemic was the duration of the succeeding waves, a key factor that could have been employed to implement public health containment strategies.

**Figure 1:**
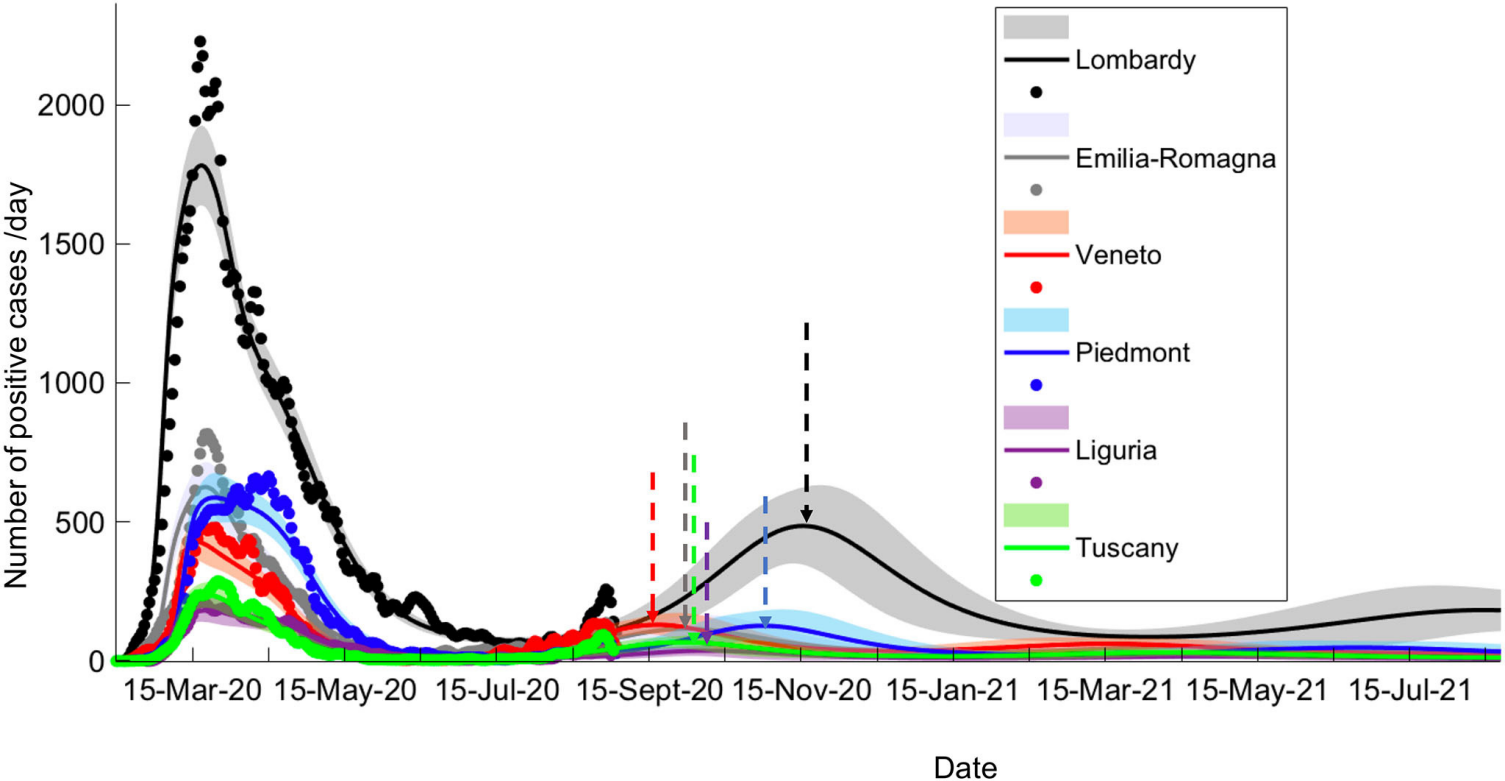
Long-term prediction following BMC. Comparison of data fitting and prediction for the six regions under consideration, obtained *via* a BMC of 32 models for each region on dataset 2 (similar results could be obtained with dataset 1; see Methods for specification on datasets). Dots represent data on daily positive cases reported by the “National Civil Protection Agency.” Data are averaged with a sliding window of 7 days. The lines reproduce posterior expectations with corresponding 90% Bayesian confidence bands (shaded). Note the different timings of the peak of the second wave and its relative intensity in the different regions. Vertical dashed arrows indicate the peak of the second wave for each prediction.

To examine the dynamics of the pandemic, we have explored how a variable period of being shielded from infection would affect the temporal evolution of the pandemic (see Methods). Using a Bayesian model comparison (BMC) procedure (see Methods, Supplementary Figure 1, left), we attempted to estimate the most probable period of being shielded from infection. To account for different periods of observation, the procedure was performed with two datasets (see Methods) and in both cases, a period of being shielded from infection of 6 months was estimated (Figure 1), resulting in an upsurge of pandemic compatibility with the actual second waves recorded throughout northern Italy. This estimate accounted for the occurrence of second waves (Figure 1), with different onset times and intensity in each region.

### Latent Causes and Model Validation

**Figure 2:**
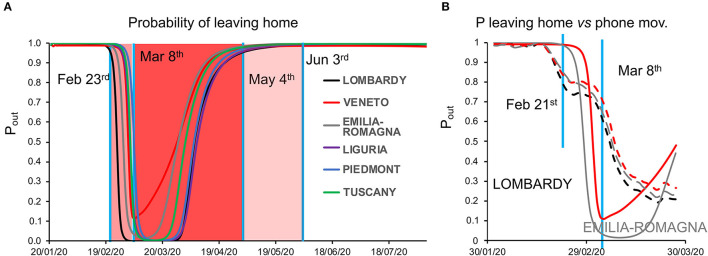
**(A)** Dynamics of the probability of leaving home. The plot shows the time course of the probability of leaving home inferred by model inversion (colored lines) in the temporal window from January 22 to August 22, 2021. During this period, the Italian government imposed a lockdown with different degrees of severity from February 23 (soft lockdown: country-wide closing of schools, universities, and all non-essential industrial and commercial activities; limiting the activities of public offices) to March 8 (tight lockdown: prohibition of any kind of mobility, apart from specific health or professional needs). May 4 is the date where the tight lockdown was relaxed and people were allowed to freely move within their region, while on June 3, people were allowed to freely move within the whole country. Vertical blue lines indicate the dates of lockdown, whereas the shaded red and pink areas represent the lockdown time windows. Note the peculiar behavior of Veneto (red line), showing a lesser reduction in the probability of leaving home, indicating a reduced adherence to the lockdown orders. **(B)** Dynamics of the probability of leaving home, compared with cell phone movements. The graph shows the trajectory of the probability of leaving home inferred by the model (solid lines) and the monitored cell phone movements (dashed lines) for the three most hit regions (Lombardy, Veneto, and Emilia-Romagna) in the period from February 21 to March 27.

Model inversion (Supplementary Figure 1, right) provided posterior estimates of the parameters (Supplementary Table 1) driving the pandemic spread and highlighting differences between regions (Supplementary Table 2; see also Supplementary Figure 2 for regional differences in the evolution of the pandemic). To evaluate the capacity of the model to correctly infer such parameters, we examined the time course of the model-estimated probability of leaving home *vis-à-vis* the known dates of the lockdown progressively imposed by the Italian government (from “soft” on February 23 to “tight” on March 8): we observed indeed a close match between the time courses (with Veneto showing a slightly different behavior from other regions), although the smooth time course output by the model obviously could not match perfectly the sharp discontinuities of the lockdown timeline (Figure 2A).

As an additional validation, we compared the probability of leaving home to the actual cell phone movement data in the temporal window from January 31 to March 31, obtaining a strong correlation, with a lesser reduction in mobility for Veneto compared with Lombardy and Emilia-Romagna [Figure 2B and Supplementary Table 3; see also (1)].

**Figure 3:**
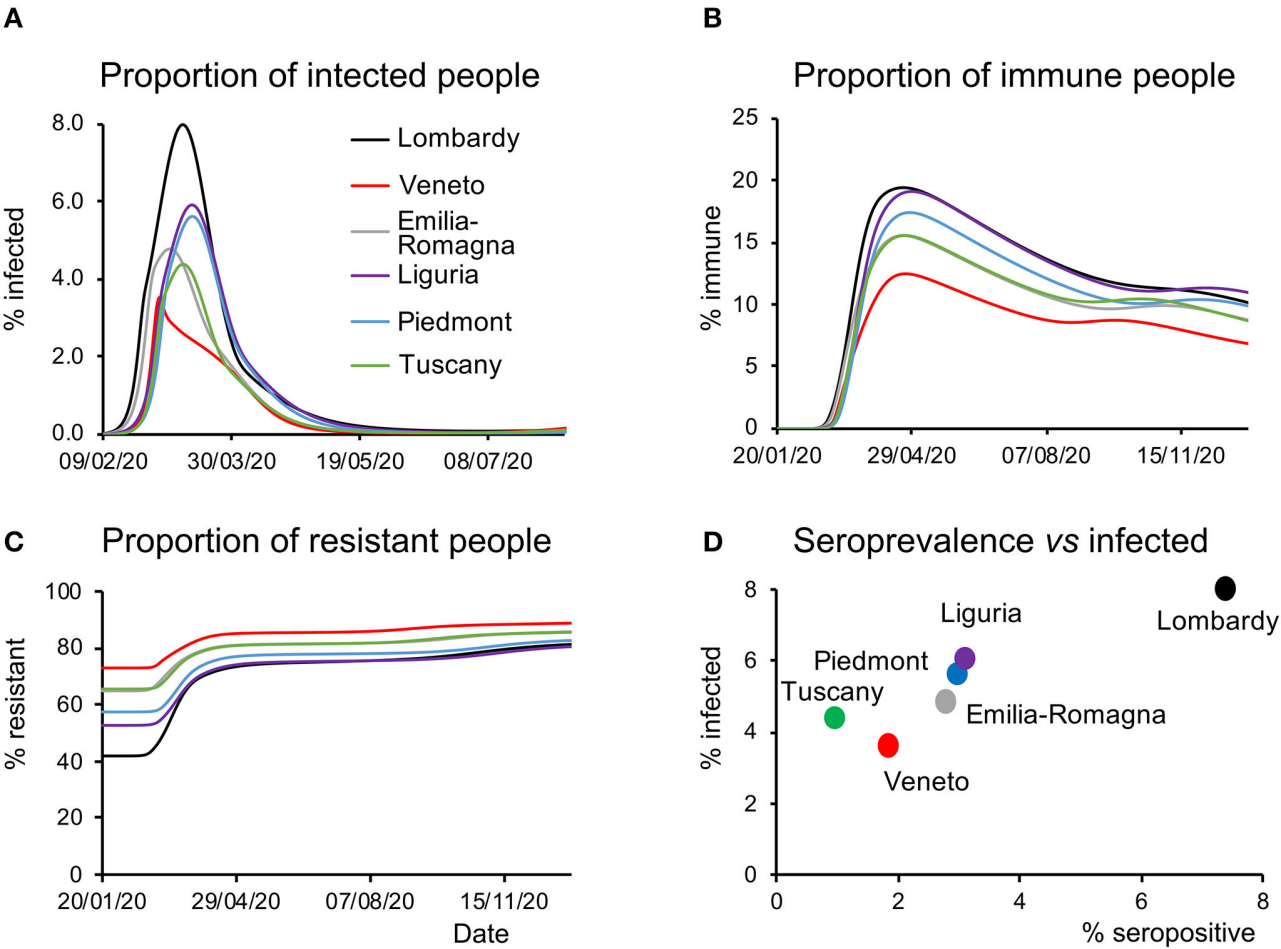
Latent causes. **(A)** Time course of the proportion of infected people during the epidemic and in the following months for all the regions considered. Note that Veneto shows a steep rise at the beginning of the epidemic but quickly drops to the initial values, whereas other regions display slower kinetics. **(B)** Time course of the proportion of immune people during the epidemic and in the subsequent months for all the regions considered. As in the case of infected people, this proportion is the lowest in Veneto throughout most of the considered time window. **(C)** Time course of the proportion of resistant people during the epidemic and in the subsequent months for all the regions considered. Notice how at the beginning of the epidemic this proportion was highest in Veneto. **(D)** Data on the serological test (% of the population that have been infected) are plotted against the peak value of the proportion of infected people inferred by the model and shown in **(A)**.

The latent causes generating the dynamics of the pandemic are related to the states within each of the four factors in the LIST model (see Methods). Focusing on the infection factor, the model-inferred proportion of infected people showed a rapid rise during the first weeks of the epidemic, peaking variably from region to region (Figure 3A), with Lombardy showing the highest and Veneto the lowest prevalence. Similarly, the model-inferred proportion of immune people rose quickly in the first weeks of the epidemic peaking with different values in each region, with Veneto and Lombardy at the bottom and top of the range, respectively (Figure 3B). Of note, the proportion of susceptible individuals begins to gradually rise back toward the initial value as (effective) immunity weakens after the first wave (Figure 3B). Conversely, the proportion of resistant individuals (see Methods) rises in response to the accumulation of people leaving the susceptible state (Figure 3C). We point out that the effective immunity (see Methods) must not be intended only as a biological parameter accounting for antibodies and T-cells-dependent responses triggered by SARS-CoV-2 infections, it rather accounts also for non-biological factors such as self-isolation and non-pharmacological measures to prevent infection.

A third validation of the model was provided by serological data. At the end of May, the Italian government launched a campaign to randomly sample the population and test whether they had been previously infected. The proportion of infected people inferred by the model (Figure 3D) correlated with the results of the serological tests (*R*^2^ = 0.92, linear regression across regions). It should be noted that the proportion of infected people inferred by the model accounts for subjects who have contracted the virus and includes in a probabilistic way, both the tested and untested cases. This is a remarkable aspect of predictive validity because these serological data were never used to inform the model parameters. Nonetheless, it should be noted that, although the model over predicts from a quantitative point of view the serological survey data, the differences in absolute value between the measures and the estimates were larger in regions with a low percentage of positive cases. The estimates of the model therefore probably reflect a low signal to noise ratio in regions with a small percentage of a positive case.

### Forecasting the Dynamics of the Pandemic

Given the matching between the inferred latent causes and empirical data, we have tested the capability of the model to generate long- and mid-term predictions, which expressed the occurrence of waves of infection in all the considered regions, with varying onset from mid-September to late November. This prediction is in accordance with the recurrence of infections observed in the Autumn 2020 (Supplementary Figures 3–6), throughout Italy. The early peaks appear in fact to be well-predicted in Veneto (mid-September) and Tuscany (early October), regions that witnessed a rise in the number of positive cases in those periods (see Supplementary Figures 5, 6). Interestingly, when data related to the early upsurges in July were withheld from model inversion (Dataset 1, see Methods), a second wave could still be predicted, albeit with a later onset (Supplementary Figure 7).

One common metric of viral transmission is the reproduction ratio (R_t_). The model-inferred value for the R_t_ did follow the timeline of the lockdown policies that mitigated viral transmission (Supplementary Figure 9). Importantly, in July, this value raised again above 1, as confirmed by official data released by the Italian Minister of Health and preluding the rise of second waves.

**Figure 4:**
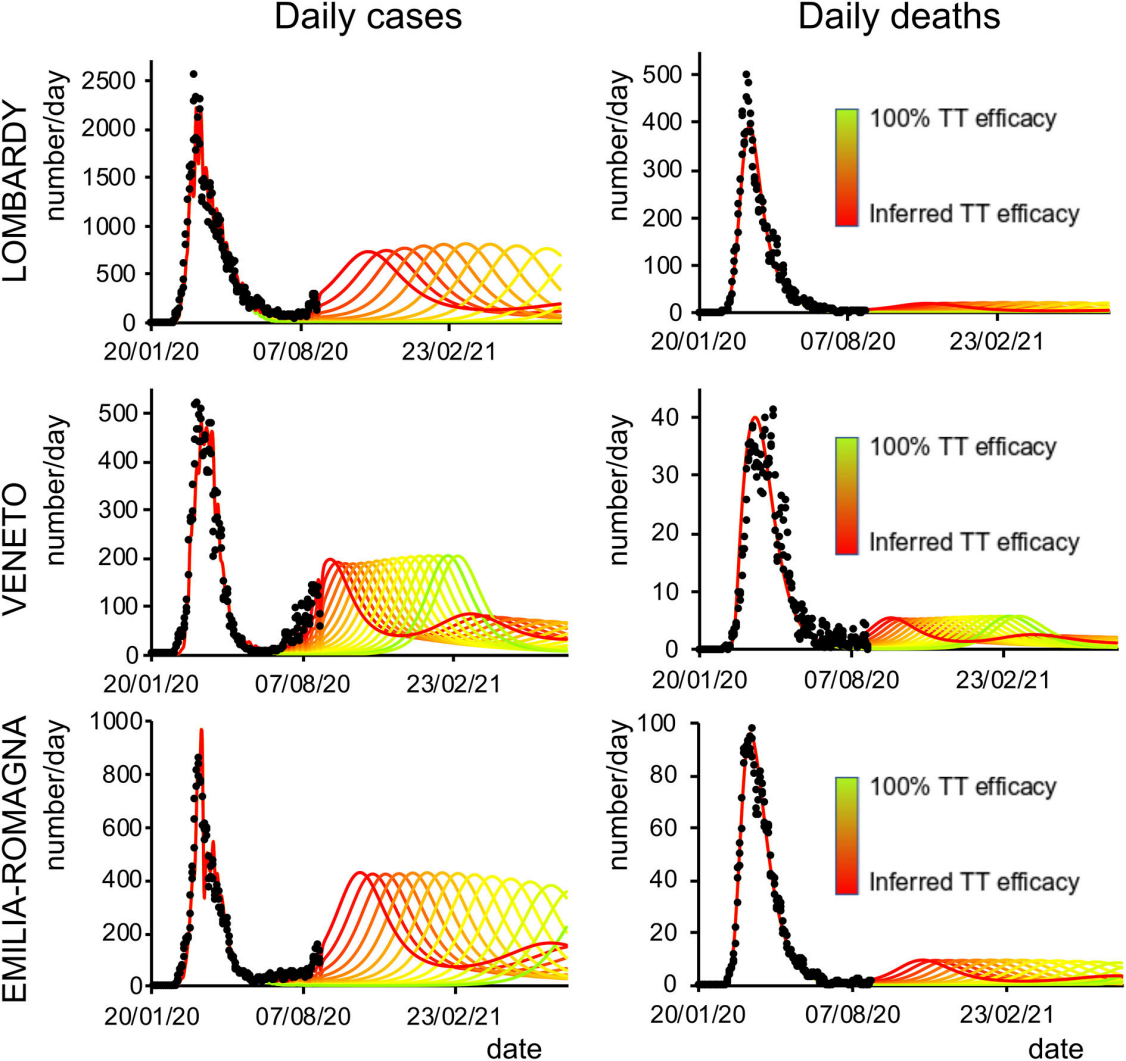
Second-wave forecasts (Lombardy, Veneto, and Emilia-Romagna): Predicted number of daily positive cases **(left)** and deaths **(right)** under increasing levels of testing and tracking efficacy. The curves go from the value inferred by the model (red line) to 100% efficacy (green line). Black dots represent daily data averaged with a sliding window of 7 days.

**Figure 5:**
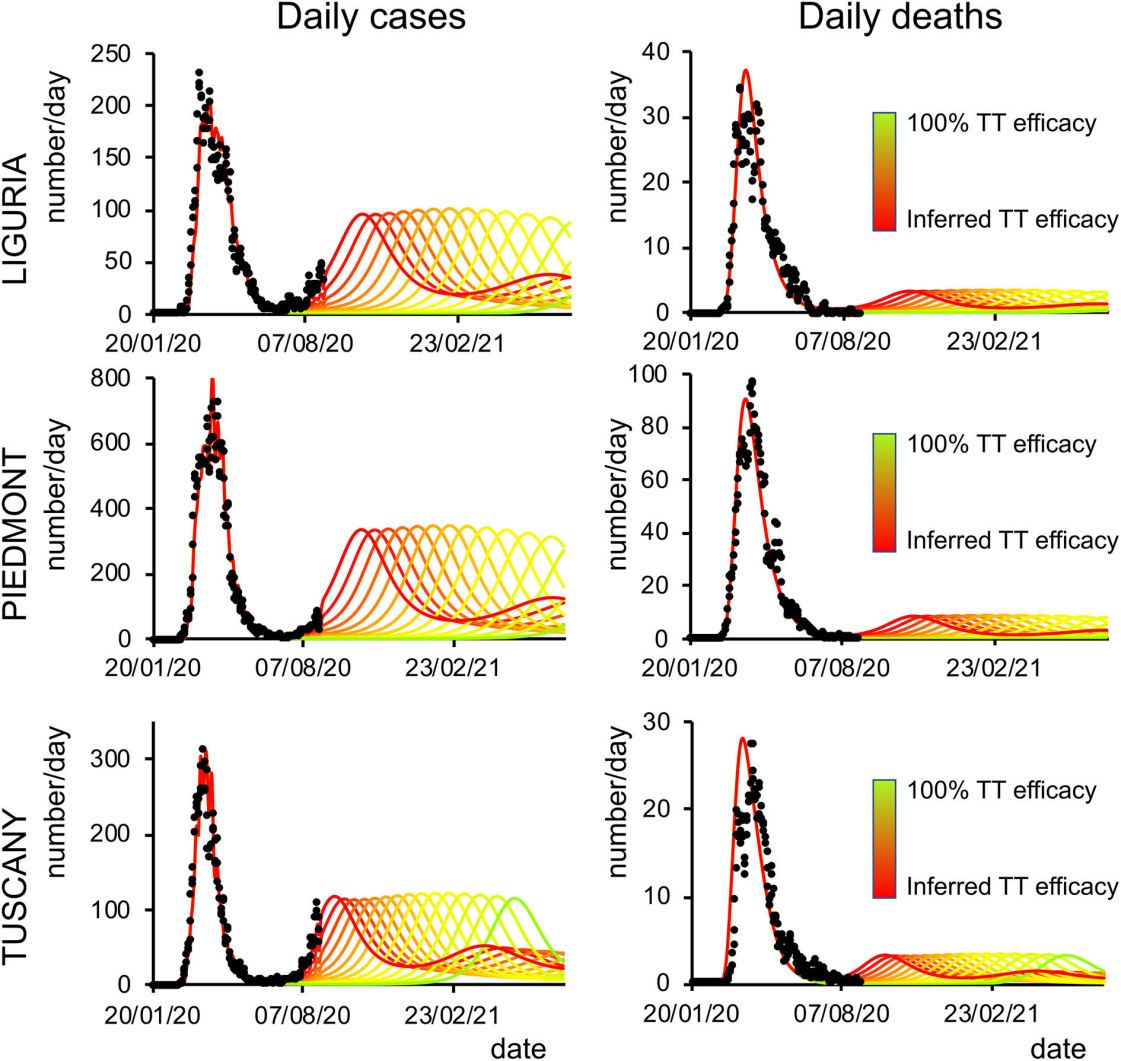
Second-wave forecasts (Liguria, Piedmont, and Tuscany): The format of this figure follows that of Figure 4.

**Figure 6:**
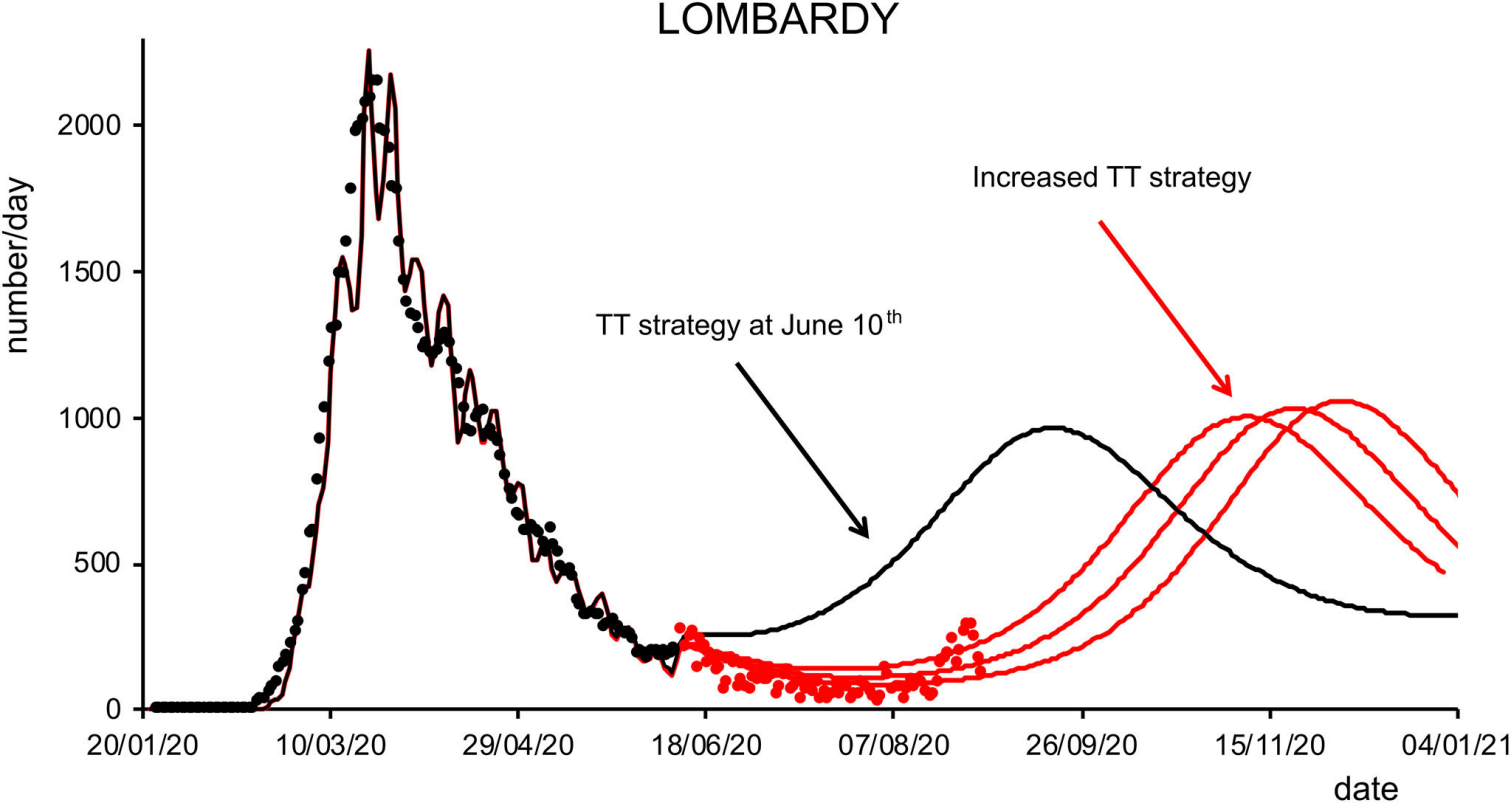
Effects of testing and tracking strategies in Lombardy. Black dots are daily data from January 22 to June 10 (7-day sliding window averages) of positive cases reported by the “National Civil Protection Agency” for Lombardy. The black line represents the posterior expectation for the same quantity following model inversion. Colored lines represent the predicted number of positive cases under increasing levels of testing and tracking, starting from the level that the model inferred to be the one adopted on June 10, to 12% (first red trace), 18% (middle red trace), and 24% (right red trace) of the maximum efficacy (see Methods). Red dots represent daily data from June 11 to August 31 (7-day sliding window averages).

Finally, we analyzed how the efficacy of testing and tracking strategies mitigated the spread of SARS-CoV-2. One of the most effective mitigation strategies in facing the pandemic is to test for infected but asymptomatic subjects and trace their contacts to contain novel outbreaks (19–22). The model can evaluate a range of possible scenarios by varying the efficacy of TTS. In particular, the efficacy of TTS was increased, in 16 equal steps, from the level initially inferred by the model to the full efficacy (i.e., testing and tracking of every asymptomatic individual). The ensuing predictions (Figures 4, 5; Supplementary Figure 10) for all regions show the occurrence of the second wave in the autumn, with a significant reduction in prevalence and fatality as TTS efficacy is increased. Notably, the model predicted that the occurrence of a second wave could have been postponed by a suitable enhancement of TTS, and this forecast is supported by the available data. As a case example, we have analyzed changes in TTS efficacy that were actually implemented in Lombardy at the end of the first acute phase of the pandemic diffusion (which the model infers to be in the range of 12–20% of the maximum efficacy). The strategy adopted by this region appears indeed to have been successful in postponing the peak of the second wave (Figure 6). Furthermore, the increase in TTS efficacy can markedly reduce the total number of deaths and positive cases (Supplementary Figure 11).

## Discussion

Dynamic causal modeling, a modeling approach originally conceived to characterize brain network dynamics, was used to infer the latent causes underlying the progression of COVID-19 in northern Italy. The effective population (i.e., the population potentially exposed to the virus) was smaller than the total (census) population size and varied over the different regions (Supplementary Table 4). This value, which takes into account individuals that can get in contact with contagious people and thus contribute to the outbreak, could indeed reflect different geospatial factors, the territory, lifestyle, behavior, and social habits. For example, Lombardy (90% effective population) presents a huge metropolitan area, where people usually share crowded means of transportation and working places. Similarly, Piedmont has most of its habitants living in the metropolitan Torino area (30% of the total regional population), one of the biggest Italian cities. Conversely, the other four regions have smaller metropolitan areas. Lombardy has more than two times the number of towns with >20K inhabitants compared with Tuscany (effective population ~30% of the census population). All these factors could have favored aggregation and viral spread, especially in the initial phase of the pandemic. In the Testing model (see Methods), where the total census population is considered, different behaviors, social habits, and geographical features affect the proportion of resistant people.

Infection, location, and clinical parameters were similar in the six regions under consideration (23, 24), supporting the notion that social and healthcare strategies were comparable and that, despite potential differences in climate and geographic factors, the virology is substantially equal. In summary, a larger susceptible population may lie at the origin of the higher impact of COVID-19 in Lombardy compared with Veneto and Emilia-Romagna, which were simultaneously hit by the outbreak. This may in turn reflect a combination of causes including geographical segregation of the population, lifestyle, social habits, and environmental factors such as air pollution and climate conditions, that may favor the virus persistence and thus individual exposure (1, 25).

Interestingly, the model inversion uncovered peculiar values for the testing parameters of Veneto, in line with the more effective prevention policies adopted by this region since the beginning of the outbreak, which included testing both symptomatic and asymptomatic subjects, while in other regions, only symptomatic cases were investigated (26, 27). This testing policy seemed to have reduced the virus spread in Veneto, as reflected in the lower pandemic toll compared with other regions (28). It is likewise interesting that the proportion of the resistant population is similar (~50%) for all regions except Veneto (60%). This difference could reflect a higher initial level of immunity determined by an early circulation of the coronavirus, as recently reported (29).

The model validation by comparison with the available epidemiological data showed that the changes in mobility inferred by the model matched both (i) the timeline of the lockdown enforced by the government and (ii) the actual mobility as tracked through cell phones; furthermore, the inferred probability of being infected was correlated with data from serological tests. It is noteworthy to point out that while the probability of leaving home for Lombardy remains at the minimum value throughout March, the probability of leaving home for Veneto, on the other hand, does increase rapidly after March 8. The trend for all other regions displays an intermediate tendency. In general, it should be noted that the probability of leaving home is a transition probability modeled as the propensity to leave home and therefore expose oneself to contacts. This propensity was inferred as a latent cause markedly determining the number of new cases and is unlikely to match exactly the actual movements, which are likely influenced by other additional factors. Furthermore, the available data of monitored cell phone movements were limited to March 30, 2020. It is quite likely that these movements would have shown an increasing trend in April, thus reducing the apparent divergence between prediction and data from March 30 onward in the graph. In particular, several of the major industrial sites, due to their crucial role for the local and national economy, resumed their activities in April and required their employees to physically go back to work. Finally, it should be noted that cell phone data only reflect movements beyond a 2-Km span and thus underestimate the actual mobility. If people started to go out more and more in the vicinity of their home before the end of the lockdown, for example, neighborhood walks became very popular to counteract the psychological repercussions of reclusion, this would have caused an upward trend in mobility within the lockdown window that could potentially have been picked up by the model but would have not been reflected in the cell phone data.

The model was also validated through a procedure typical in machine learning (see Methods and Supplementary Material). Whereas, there was overall a good agreement between model and data when using dataset 1, some points (positive cases) did fall outside the credible intervals when considering dataset 2 (Supplementary Figures 3, 4). It is important to note that, after June 30, sudden infection foci were related to episodic and isolated outbreaks in confined areas. This may be explained as follows: (i) traditionally, the Italian population moves around *en masse* during late July and August for vacation; (ii) although, in 2020, international tourism was markedly reduced, Italy is traditionally one of the preferred destinations and thus remained exposed to imported cases; (iii) most of the cases observed in the late summer were related to Italian tourists coming back from foreign countries, where infections were increasing; (iv) in summer, Italy is particularly exposed to migrants, with no exception in 2020. Migrants, when intercepted, are screened and quarantined if needed, but some probably escape the entry filter and may propagate the infection.

By comparing actual and predicted data quantitatively, the model prediction values appear to be about 10 times smaller than data obtained from the repository of the “Italian Civil Protection” (around 800 vs. 8,000 daily positive cases at the peak of the second wave). A potential explanation for this discrepancy lies in the fact that predictions have been generated using data on daily positive cases and daily tests, whereas the policy of tests administration on the regional population has substantially changed during the pandemic evolution. During the early phase, pharyngeal nasal swabs were performed mainly on symptomatic and hospitalized people whereas, during the late phase, tests were performed extensively on a larger number of subjects. The result is a collectively larger number of performed tests and a lower percentage of symptomatic vs. positive tests. For instance, the ratio between the number of daily hospitalizations and daily tests during the first wave in Lombardy was on average 41 ± 3.3% (mean ± SD; range from 52% on February 25 to 18% on March 25) and 4.4 ± 0.1% (mean ± SD; range from 4.5% on October 10 to 3.5% on November 9) during the second wave (see Supplementary Figure 13), which amounts to roughly to a 10-fold decrease. Conversely, but in a similar 10-fold proportion, the total number of tests performed daily massively increased from the first wave (3,127 ± 284, mean ± SD; in the period between February 25 and March 25) to the second wave (3,4220 ± 1,160, mean ± SD; in the period between October 10 and November 9). We can speculate that the increase in the number of performed tests while affecting the absolute amount of detected infections, did not change the dynamics of the diffusion which were indeed correctly predicted (Supplementary Figure 14).

The model predictions for the COVID-19 pandemic entail the occurrence of second waves in the geographical areas under the study. The characteristics of the forecast depend on whether the simulation uses data excluding (dataset 1) or including (dataset 2) the movement-intensive months of July and August (Supplementary Figures 3, 4 vs. Supplementary Figures 5, 6; Supplementary Figure 7). We decided to limit the temporal window of observation to November 2020 rather than also including the first months of 2021 since virus variants with different infective properties that could alter the dynamics of the pandemic were first detected at the end of 2020. Lombardy showed small differences in the predicted number of cases and deaths using the two datasets, while Veneto, Piedmont, and Tuscany, due to a sharp increase of cases in the first weeks of August, showed marked differences in the onset of the second wave when using datasets 1 or 2. Indeed, those cases were mainly related to an increase in holiday-related population fluxes among regions or, alternatively, because of immigration, both seasonal events that could not be predicted by the model at that stage (the model could in principle predict seasonal trends if multiyear data are used for model inversion). Furthermore, the evaluation of the impact of different strategies of healthcare policy (Figures 4, 5), mainly by increasing the efficacy of TTS (see Methods), shows that they can influence the occurrence of the second wave and reduce its impact in terms of lives saved and pressure on the healthcare system. In all cases, the severity of the second wave predicted by the model is markedly lower than the one of the first wave in terms of deaths, the proportion of infected people, and proportion of people showing symptoms as it was indeed observed in northern Italy (see Supplementary Table 5).

The proposed study, despite the advantages afforded by DCM, suffers from some limitations, which have already been mentioned in the original model development (17): (i) the model does not consider interactions with seasonal flu or other annual fluctuations (30); (ii) as with other modeling approaches, the outcomes of the BMC and posterior inferences are strictly model dependent; (iii) updating the model with available data could change the posterior predictions; (iv) the model does not include geospatial aspects, but rather each outbreak is treated as a point process (31); and (v) the accuracy of the model inversion and the subsequent predictions depend on the amount of the available data, being proportional to the percentage of positive cases.

Nevertheless, with reference to the above listed points, it is important to consider that: (i) annual fluctuations such as seasonal flu can alter the overall statistics but are uniformly distributed throughout the country without preferential geographical localizations, and therefore, the potential error is systematic and homogeneously distributed; (ii) the outcomes of every model are limited to the approach that is actually employed (i.e., this is not a specific shortcoming of DCM); (iii) the continuous updating of the model and therefore of the posterior predictions is inherently one of the main advantages of this kind of approach and could thus be regarded as a feature rather than as a flaw. The flexibility of the model takes into account unexpected and sudden events that can promptly change the pandemic dynamics allowing therefore to update the prediction generating different future scenarios; (iv) once more, treating each outbreak as a point process provides the model with the capability to interpret as potentially dangerous both single and spotted events, without the need to collect all the data to yield a uniform prediction; (v) newly available data can be used to improve the accuracy of the prediction.

In conclusion, the retrospective analysis of the latent causes of the pandemic enables the uncovering of factors that can be effectively controlled by institutional and healthcare policies and provides an effective procedure to simulate the impact of the latter on second-wave scenarios. This should allow better planning by optimizing the benefit-cost ratio of such policies in view of the specific means and resources available to each region, for example, by balancing strategies such as lockdown enforcement, testing, and tracking protocols, and vaccinations.

## Methods

### Data Sources

The data on the pandemic evolution were obtained from the official repository of the “National Civil Protection Agency” (https://github.com/pcm-dpc/COVID-19). Specifically, we used the daily number of confirmed positive cases, deaths, and recovered cases from January 22 to August 31. This dataset was split into two subsets (dataset 1: from January 22 to July 20; dataset 2: from January 22 to August 31) to accommodate differences in the social and movement patterns before and after the end of July, a period when the great majority of the Italian population leaves home for the summer vacations. Data on daily individual movements were collected anonymously *via* mobile cell phone networks (1). Based on the position of cell phones in the six investigated regions, as available through the call detail records (CDR) of SIM card data of around 27 million Italian residents, we computed the regional daily number of cell phone movements, weighted by the provincial population (only changes in cell phone position >2 Km were considered). The data on the seroprevalence of SARS-CoV-2 antibodies were made available by the Italian Ministry of Health (http://www.salute.gov.it/portale/news/p3_2_1_1_1.jsp?lingua=italiano&menu=notizie&p=dalministero&id=4998), as identified through a serological population-based survey performed by the “Italian Croce Rossa” from May 25 to July 15.

### Correlational Analyses

To evaluate the capacity of the model to infer latent variables, we assessed how well the probability of leaving home tracked the time series of cell phone movements in the latest period available (from February 1 to March 27), *via* a Pearson's correlation analysis.

### The Dynamic Causal Model

The approach adopted in this paper is based on the work of Friston and colleagues developed during the first outbreak of the pandemic (March–July 2020). In particular, we refer to “(16); *Testing and tracking in the UK: a dynamic causal modelling study”* and “(17); *Effective immunity and second waves: a dynamic causal modelling study.”*

In the following section, we summarize the basic principles of the theoretical framework of DCM applied to the COVID-19 pandemic.

#### The Basic Structure of the Model and Its Applications

The DCM methodology (13) has been recently applied to the worldwide COVID-19 diffusion based on an epidemiological compartmental model, readers are referred to the original technical reports for details (14–17). In summary, the requisite generative model rests upon a mean field approximation to population dynamics, where individuals can be probabilistically characterized by their state within four factors, representing respectively their *location, infection, symptoms*, and *testing* state (LIST model, Supplementary Figure 1). These factors are coupled through probabilistic transitions specified by state probability transition matrices (for an example, the transition matrix for the location factor in “*Model parameters and latent causes*”). There are 26 model parameters in total, which parameterize the probabilities of occupying each state and transitioning between states. These parameters are initialized with *a priori* expectations and variances (see Supplementary Table 1). The DCM model inversion maps from the observed data to the estimated parameter values using gradient ascent until the marginal likelihood of the data (aka, model evidence) is maximized. Technically, the gradient ascent is on a variational bound on model evidence, known as evidence lower bound (ELBO) in machine learning, and variational free energy in physics. The posterior densities of the parameters returned by the procedure can be used to simulate the impact of various parameters on the dynamics of the process.

We adopted the same approach to model the pandemic evolution in the six Italian regions showing the maximum number of deaths during the first 3 months of the outbreak, namely, Lombardy, Veneto, Emilia-Romagna, Liguria, Piedmont, and Tuscany. More specifically, we used DCM for the following aims (Supplementary Table 5):

1) *Verifying the Predictive Ability of the Model*

Initially, we assessed the model's forecast against the actual data. To do this, we obtained the posterior estimates of model parameters (Supplementary Figures 3–7) based upon a subset of the available data, namely, the number of daily positive cases, deaths, and recovered cases from January 22 to June 30 (dataset 1) and from January 22 to August 11 (dataset 2) and compared the model forecast to the actual data for the time points from June 30 to July 20 (dataset 1) and from August 12 to August 31 (dataset 2). The first temporal window was chosen so as to exclude, for the purpose of model validation, the last week of July. Traditionally, the great majority of Italian people leave home for vacation at the end of July or the beginning of August, marking a period with significant changes in social interactions and movement patterns that could not be predicted by the model. The second temporal window was added in an attempt to include as much of the data corresponding to the vacation fluxes as possible at the time of writing the manuscript.

2) *Estimating the duration of immunity and its impact on the expected onsets of second waves*

For this purpose, we used BMC, as described in (16). The BMC consists in comparing models differing in latent causes parametrization to evaluate changes in model evidence. This procedure allows identifying which latent causes subtending mechanistic hypotheses are more explicative of the collected data. In our case, the BMC was performed on models differing for the rate at which the effective immunity is lost.

A total of 32 DCM models were specified with data comprising the number of daily positive cases and deaths (Supplementary Figure 1A) on the time interval from January 22 to August 31 (dataset 2, see Methods). Each model differed only in the prior assumption about the duration of immunity, from 1 to 32 months in monthly increments. The model evidence for each of the 32 models was pooled over the six Italian regions under consideration, yielding a marginal likelihood for each potential duration of being shielded from infection (“effective immunity”). It is important to note that, as explained in the section “*Model parameters and latent causes,”* the period of immunity is not a hard threshold denoting a sudden loss of immunity, rather it represents the time constant (τ) of an exponential waning of immunity, which can depend on several factors such as population fluxes that can change the size of the susceptible pool, or factors depending on virus and host [see (17) for a detailed explanation]. Since the loss of protection from the infection is a crucial factor in determining outbreak recurrences, its posterior estimate was used to forecast the onsets of second waves in the various Italian regions.

3) *Evaluating the effect of varying degrees of the efficacy of the testing and tracking strategy on second-wave outbreaks*

Different aspects of testing and surveillance were analyzed through the testing and tracking procedure (16). The posterior estimates of model parameters (Supplementary Figure 1B) are based upon dataset 2 on the number of daily positive cases, deaths, and performed tests in all the six Italian regions under consideration. Crucially, the total number of tests allows a more informed parameter estimation (see Supplementary Figure 2). The efficacy of testing and tracking policy is defined as the probability that a subject will be offered a test if infected and asymptomatic (16). Efficacy varies from 0%, where the probability of being offered a test when infected and asymptomatic is null, to 100% if the subject will certainly be tested and subsequently self-isolated. We performed a series of simulations by incrementing in 16 steps the testing and tracking efficacy from 0 to 100%. For the purpose of the simulation, the testing and tracking strategy was assumed to be introduced 20 weeks after the first outbreak.

#### Model Parameters and Latent Causes

The model we used is formally identical to the one described in (16, 17) and the full list of model parameters can be found in Supplementary Table 1. Here, we describe in greater detail the hidden states that are particularly relevant for the present purposes (“hidden” means that these quantities were not directly observed but were *inferred* by the model):

- The *probability of leaving home* (location factor) given the condition of being asymptomatic has a prior baseline value (see “probability of going out” parameter in Supplementary Table 1) multiplied by a decreasing function of the proportion of infected people. The social distancing, modeled as “the propensity to leave home and expose oneself to interpersonal contacts (14),” is an exponential threshold parameter (see social distancing threshold Supplementary Table 1). The transition probabilities among the location factor can be defined for each state of the clinical factor as follows: the probability of being in a certain location arriving from a different location is regulated by:
  $$\begin{array}{l} P=\;{\theta_{out}\left(1-p_{infected}\right)}^{\theta_{sde}} \end{array}$$

Where θ^*out*^ is the probability of leaving home normally every day, θ^*sde*^ is the social distancing threshold, and *p*_*infected*_ is the probability of being infected. For example, the probability of being asymptomatic in a certain location is defined by the following (5 × 5) transition matrix


$$\begin{array}{l} P\left(loc_{t+1}|loc_{t},clin_{t}=asymptomatic\right)=\\ \left(\begin{array}{ccccc} home & work & CCU & morgue & isolation\\ \left(1-\theta^{out}\right) & 1 & 1 & \left(1-k_{day}\right) & \left(1-P_{iso}\right)\\ \theta^{out} & 0 & 0 & 0 & 0\\ 0 & 0 & 0 & 0 & 0\\ 0 & 0 & 0 & {\;k}_{day} & 0\\ 0 & 0 & 0 & 0 & P_{iso} \end{array}\right) \end{array}$$

where the columns indicate the current location, and the rows indicate the next location. In all the transition probability matrices of the location factor, rows, and columns are ordered as *home, work, CCU, morgue, and isolation*. The parametrization of transition probabilities is in terms of rate parameters (such as $\theta^{out},\;{\;k}_{day},\;P_{iso}$) as in (14).


$$\begin{array}{l} \theta =\exp\left(-k\right)=\exp\left(-1/\tau \right) \end{array}$$

where the rate parameter θ is related through an exponential decay to the rate constant κ and ultimately with the time constant τ, to indicate that the probability of staying in one state is determined by the typical occupation time of that state. For instance, the parameters *P*_*iso*_ can be specified in terms of the number of days people remain in self-isolation (τ = 7 *days*, *P*_*iso*_ = exp(−1/7)). The permanence in the morgue state is also transitory (τ = 1 *day*, *k*_*day*_ = exp−1) to conserve the total population since deaths are compensated by newborn susceptible individuals (17).

- The *proportion of infected people* (infection factor) represents the proportion of people who are infected at a certain time. The probability of being infected depends upon the number of social contacts and the proportion of time spent at home. These dependencies are parametrized by the “effective number of contacts being home and being out” and the “probability of getting contagion for each contact” (Supplementary Table 1).
- The *proportion of immune people* (infection factor) represents the number of people that are effectively probabilistically immune at a certain time.
- The *proportion of resistant people* (infection factor) represents individuals that are not susceptible to infection because they are relatively protected from the infection by immunity, such as crossreactivity (32, 33) or protective host factors (34, 35). Notably, during the pandemic, people can transit from a state of exposure to a state of resistance through a mild illness that does not entail seroconversion, with recovery being mediated by T-cell response (36). The resistant state also includes the immune state for subjects who never became contagious.

Similarly to what shown above, the probability of moving between different states of infections is:


$$\begin{array}{l} P=\;\left(1-\theta_{trn}\cdot p_{infectious}^{\inf}\right) \end{array}$$

Where θ_*trn*_ is the transition probability per contact.

- The *proportion of people showing symptoms* (symptoms factor) represents the proportion of subjects developing symptoms after being exposed.

In the DCM approach, the definition of these factors and the associated transition probabilities constitutes a generative model of the observable data, based on the hypothesized real (but latent) causal structure of the process under the study. Starting from the LIST model [See model scheme on the left in Supplementary Figure 1 and in Figure 3 in (15)], one of the main assumptions is that the total population is divided into two compartments: *resistant* and *susceptible:*

- The *resistant* state is an absorbing state (once entered, people stay in that state) and includes individuals that are not susceptible to infection because either they have preexisting immunity (due to crossreactivity or specific host factors) or they are shielded from the virus by virtue of geographical segregation. Some susceptible people who have been exposed to the virus can enter the *resistant* state without entailing seroconversion since the recovery is mediated at a cellular level (T-cells).
- The *susceptible* state includes people that can be infected and potentially undergo seroconversion or alternatively, through a mild illness, can enter the resistant state without seroconversion (see above) since they show innate immunity.

The quota of susceptible individuals that can be temporarily shielded from the infection was modeled *via* the “***effective immunity***” parameter accounting for the prevalence of antibodies against coronavirus and lifestyle factors such as isolation due to mitigation strategies. The ***effective population immunity*** parameter represents therefore the proportion of people who, albeit potentially susceptible, cannot contract or transmit the virus at a certain point in time. The effective immunity has been assumed to decay due to antibodies decay (37), virus mutation (30, 31), or fluxes of people that can dilute the preexisting immunity (38).

The results were obtained using MATLAB code available as part of the free and open-source academic software SPM (https://www.fil.ion.ucl.ac.uk/spm/), released under the terms of the GNU General Public License version 2 or later.

#### Model Predictions Using Tests as Input Data

To analyze the impact of different testing and tracking strategies, we used a version of the model that received as input, in addition to the daily number of positive cases and deaths, also the daily number of positive tests (Testing model, Supplementary Table 6). This procedure increased the data fitting accuracy (see Supplementary Figure 2), since the number of positive cases was effectively controlled for the number of actual tests. As shown in Supplementary Figure 2, data fitting was markedly improved (compare Supplementary Figures 3, 6 to Supplementary Figure 2) and the model prediction tended to follow closely the cyclic data fluctuations.

#### Model Validation and Predictive Validity on Dataset 1

The time series of the estimated daily number of positive cases and a daily number of deaths, as compared to the actual data, are reported in Supplementary Figures 3, 4. The predictive capability of the model was assessed by withholding data from the last 20 days during model inversion (see Methods). The time series considered included data from January 22 to June 30. This choice was motivated by the customary social pattern of the Italian population to go on vacation *en masse* in a period ranging from early July to late August. We wanted to exclude this period from the forecast of the model because it entailed a sharp variation in people movements that could not be realistically predicted by the model. The model forecast was then compared with the actual data for these time points (Supplementary Figures 3, 4 zoomed views; model forecast: black lines; actual data: red dots). If we used data up to July 20, one can see that while the model predicted a slight increase for the number of daily positive cases (Supplementary Figure 7), which is arguably due to the beginning of the waning of effective immunity, inaccurate predictions are most likely due to isolated local outbreaks typically occurring during vacation periods, people returning from foreign countries after vacations or migrant fluxes. In all cases, the data related to dataset 1 fall within the credible interval of the forecast for both the positive cases and the daily deaths.

#### Predictive Validity on Dataset 2

The procedure performed to validate the model described above was repeated with data up to August 31, with 20 days starting from August 11 excluded from input data to assess the model's predictions. In this case, the model predicted an increasing trend for both the number of daily positive cases and deaths for Lombardy and Emilia-Romagna, the regions with the highest number of daily cases at the peak of the epidemic (Supplementary Figures 5, 6). The forecast for these regions attests to a mild increment of daily cases and deaths that follow the general trend of prediction and mostly remain within the Bayesian 90% credible interval. Notably, the predictions for Veneto, Piedmont, and Tuscany are expected to reach a baseline close to zero within the subsequent few weeks. The actual data (Supplementary Figures 5, 6) for the number of positive cases fall outside the 90% credible interval and may thus prelude to a sudden start of the second epidemic wave. It is however important to highlight that these data often reflect new outbreak episodes in small and localized areas, such as the one reported in a migrant reception center at the beginning of August in Veneto. It should also be noted that, in both cases, the number of daily deaths follows the general trend of the prediction, lying mostly within the confidence bands (Supplementary Figure 6).

#### Estimates of the Period of Loss of Shield From Infection

The period of immunity loss was estimated by BMC on the 32 models for each of the regions under consideration (see Supplementary Figure 8). Model inversion returned accurate data fitting for the total number of deaths and positive cases, as shown in Supplementary Figure 8.

#### Effective Reproduction Rate

The model of infection transmission supports the calculation of an *effective reproduction rate* [R_t_, always positive (16)], which represents how many people an infectious individual is likely to infect. This quantity depends upon the probability that any contact will cause an infection (*transmission strength* parameter), the probability of transition between the susceptible state and the infection state, and the number of people an individual contacts either at home or out (*effective number of contacts: home* and *effective number of contacts: out* parameters, respectively). Note that the effective reproduction rate is not a biological constant rate; it is rather a useful epidemiological value indicating the rate of outbreak diffusion. When R_t_ <1, the infection will decay to an endemic equilibrium, otherwise, the infection will spread with a velocity proportional to R_t_ (Supplementary Figure 9).

Among the various possibilities to estimate the R_t_, we have adopted the one used by (16), since the effective reproduction (R_t_) rate is not a model parameter itself, but a quantity that can be calculated from the latent causes inferred by the model according to the following equation:


$$\begin{array}{l} R_{t}=e^{\left(K_{t}\cdot \tau_{con}\right)} \end{array}$$

where


$$\begin{array}{l} K_{t}=\ln\frac{P\left(infected_{t+1}\right)}{P\left(infected_{t}\right)} \end{array}$$

The equation reflects both the growth of the proportion of infected people and the period they remain infectious (τ_*con*_).

#### Sensitivity Analysis

An example of sensitivity analysis related to the influence on death rates of the testing factor for Lombardy is shown in Supplementary Figure 12. Sensitivity is calculated as the partial derivative of the outcome with respect to a specified parameter for each timestep (sensitivity may be different at different times in the evolution of the process and it is thus represented as a time course). The change in *cumulative* deaths with respect to the specified parameter was calculated by integrating the sensitivity over the period of 100 days from January 1 to April 10 (short-term period, representing the first half of the first wave) and over the remaining part of the curve (long term).

#### Efficacy of Testing and Tracking Strategy

To quantitatively estimate the effect of testing and tracking strategies, we computed the cumulative number of positive cases and deaths in different conditions. Values were then normalized to the maximum cumulative number and plotted on a relative scale (Supplementary Figure 11).

## Data Availability Statement

The original contributions presented in the study are included in the article/Supplementary Material, further inquiries can be directed to the corresponding authors.

## Author Contributions

DG, GP, and JM designed the research and analyzed simulations. DG and JM performed simulations and customized the model. TF contributed to the overall layout of the research and to the writing. TF and MV analyzed mobility data. AG analyzed cellphone data. DG, GP, TF, ED'A, and JM wrote the first version of the manuscript. All authors contributed to revise the last version.

## Funding

This work was supported by:

– SMART-BRAIN; partnering project to the European Union's Horizon 2020 Framework Programm for Research and Innovation under the specific GA N° 785907 (Human Brain Project SGA2) to JM.
– FISR 2020 EPICELLULARCOVID19 grant by the Italian Ministry of the University and Research to MV.
– European Union's Horizon 2020 Framework Programme for Research and Innovation under the Specific Grant Agreement N° 945539 (Human Brain Project SGA3) to ED.

## Conflict of Interest

AG is employed by TerrAria s.r.l. The remaining authors declare that the research was conducted in the absence of any commercial or financial relationships that could be construed as a potential conflict of interest.

## Publisher's Note

All claims expressed in this article are solely those of the authors and do not necessarily represent those of their affiliated organizations, or those of the publisher, the editors and the reviewers. Any product that may be evaluated in this article, or claim that may be made by its manufacturer, is not guaranteed or endorsed by the publisher.
