# Supplementary material for: Modeling Early Phases of COVID-19 Pandemic in Northern Italy and Its Implication for Outbreak Diffusion
Source: Front Public Health. 2021 Dec 16;9:724362. doi: 10.3389/fpubh.2021.724362 (PMC8716563; doi:10.3389/fpubh.2021.724362)
Supplement: Supplementary file 1 [file Data_Sheet_1.pdf]

SUPPLEMENTARY MATERIAL TO THE MANUSCRIPT:

"*Modeling COVID-19 pandemic in northern Italy predicts second wave scenarios*"

Daniela Gandolfi[1†], Giuseppe Pagnoni[1,2†], Tommaso Filippini[1†], Alessia Goffi[3], Marco Vinceti[1,4], Egidio D'Angelo[5,6], Jonathan Mapelli[1,2]

[1]*Department of Biomedical, Metabolic and Neural Sciences, University of Modena and Reggio Emilia, Modena, I-41125, Italy*

[2]*Center for Neuroscience and Neurotechnology, University of Modena and Reggio Emilia, Modena, I-41125, Italy*

[3]*TerrAria, Milan, Italy*

[4]*Department of Epidemiology, Boston University School of Public Health, Boston, MA, USA*

[5]*Department of Brain and Behavioral Sciences, University of Pavia, I-27100, Pavia, Italy*

[6]*Brain Connectivity Center, IRCCS Mondino Foundation, I-27100, Pavia, Italy*

Supplementary figures

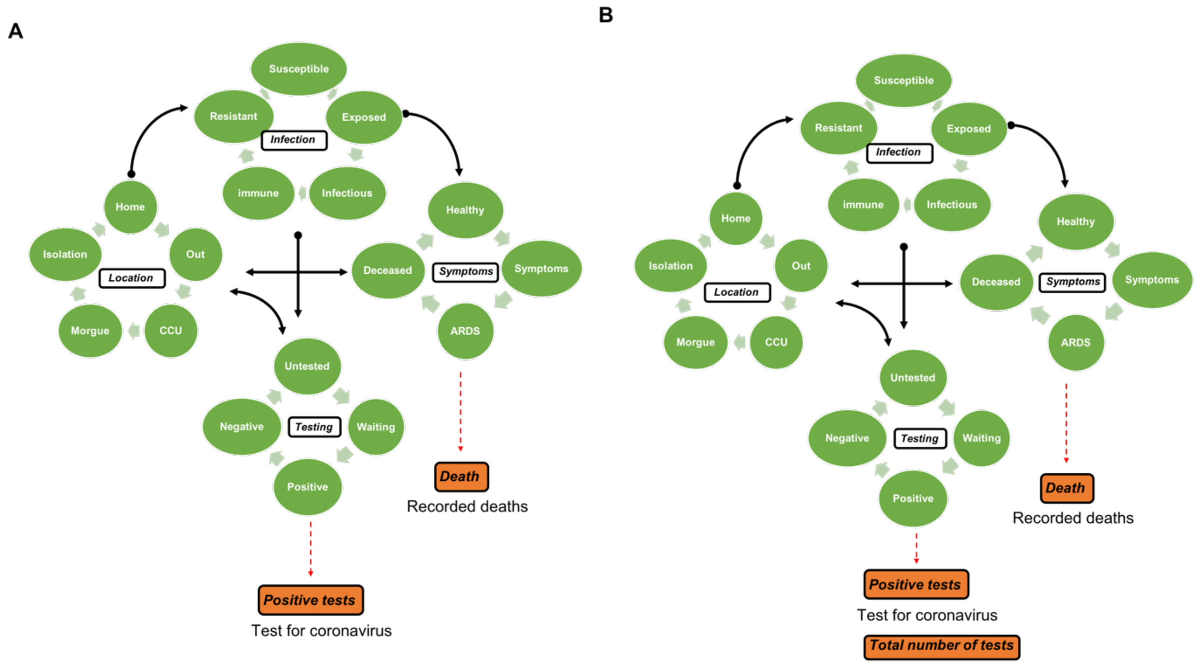

Fig SM-1 Model. The DCM model employed in this study comprises 4 distinct factors (Location, Infection, Symptoms and Testing), divided into states that characterize each

individual in a population. The states within any factor are mutually exclusive, thus every individual must be in one and only one of the states associated with the each of the four factors. The transition probabilities from one state to another depend on the model parameters, which are initially specified with prior densities and are then updated through model inversion to yield posterior estimates. In the following, we provide a brief description of each factor. **Location**. Each individual can be located i) at *home* (a low contact risk location), ii) at *work* (a high contact risk location), iii) in a *Critical Care Unit* (CCU), iv) in *isolation* or v) in the *morgue*. **Infection**. Each individual can be i) *susceptible* to being infected by virus; ii) *exposed* to the virus (the individual is in contact with infected subjects) iii) *infectious* (the individual has contracted the virus and can infect other subjects), iv) *immune* (the infected individual has acquired immunity), or v) *resistant* (this is a category of people that are shielded, by virtue of host or geographical factors, from the infection). **Symptoms.** Individuals can be i) *healthy*, ii) *symptomatic*, iii) exhibiting ARDS (acute respiratory distress syndrome), or iv) *deceased*. **Testing**. Individuals can be i) *untested*, ii) *waiting* for the test outcome, iii) *positive*, or iv) *negative* on PCR testing. The factors (white rectangles) are divided into segments (states – green disks), while the orange boxes represent the observable outputs generated by the dynamic causal models (in A, daily reports of positive tests and deaths; in B, daily reports of the number of tests performed, of positive cases and deaths. In particular, the model in A (BMC model) is parametrized with the *effective* population size, which represents people actually in contact with contagious individuals, while the model in B (Testing model) is parametrized with the total (census) population size (which includes the effective population size). (*Figures adapted from*[16,17]).

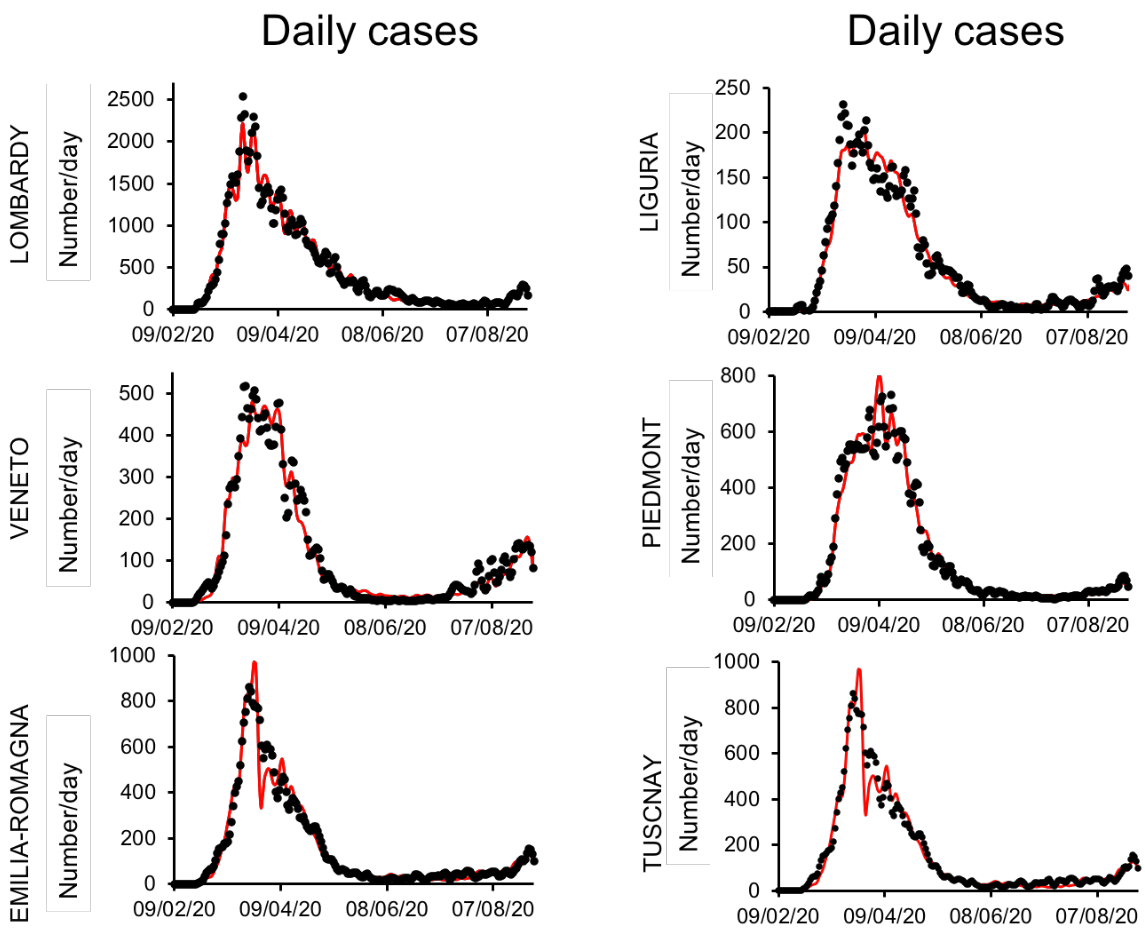

Fig. SM-2. ***Model predictions using tests as input data.*** The plots show the model fitting (red lines) to the actual data about the number of daily positive cases for the six regions under consideration resulting from the simulations performed by using the number of daily tests as input. As can be gleaned from the graphs, model fitting is significantly improved (cfr Fig 1 in the main text), even for what concerns the cyclic fluctuations (in the range from a few days to one week) related to the working period of healthcare workers performing PCR tests.

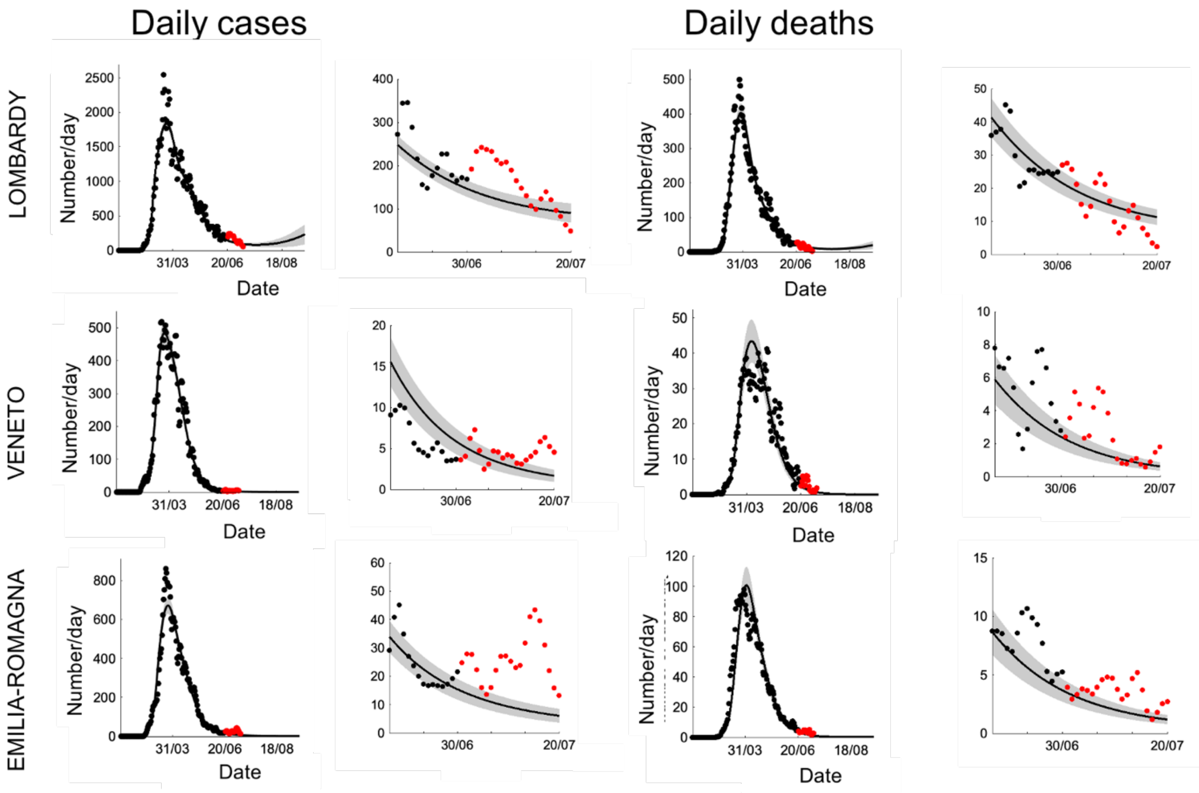

Figure SM-3: Predictive validity of the model on dataset 1 (Lombardy, Veneto and Emilia-Romagna). *Left*. Black dots represent daily data (7-day sliding-window average) of positive cases reported by the "National Civil Protection Agency" for the six regions under investigation. Black lines represent the posterior expectations following model inversion. Gray bands represent the 90% credible intervals. Red dots correspond to data of reported positive cases from June 30th to July 20th that were excluded from data fitting, in order to validate the model prediction on the time interval shown in the inset. *Right*. Similarly, plots show the reported daily deaths from January 22nd to June 30th (black dots) and the corresponding fitting with 90% credible interval (black lines and gray bands). Red dots represent daily deaths in the subsequent 20 days along with model prediction and 90% interval (inset).

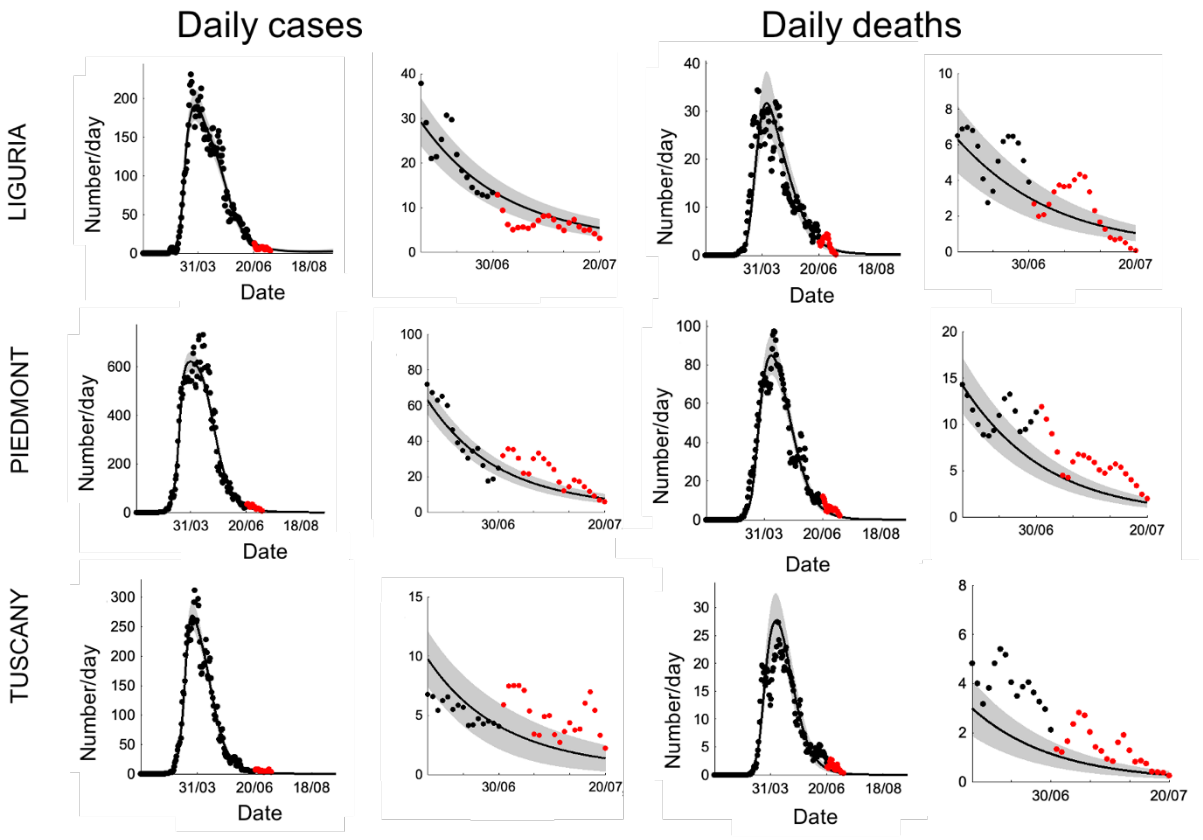

Figure SM-4. Predictive validity of the model on dataset 1 (Liguria, Piedmont and Tuscany).

This figure uses the same format as figure SM-2.

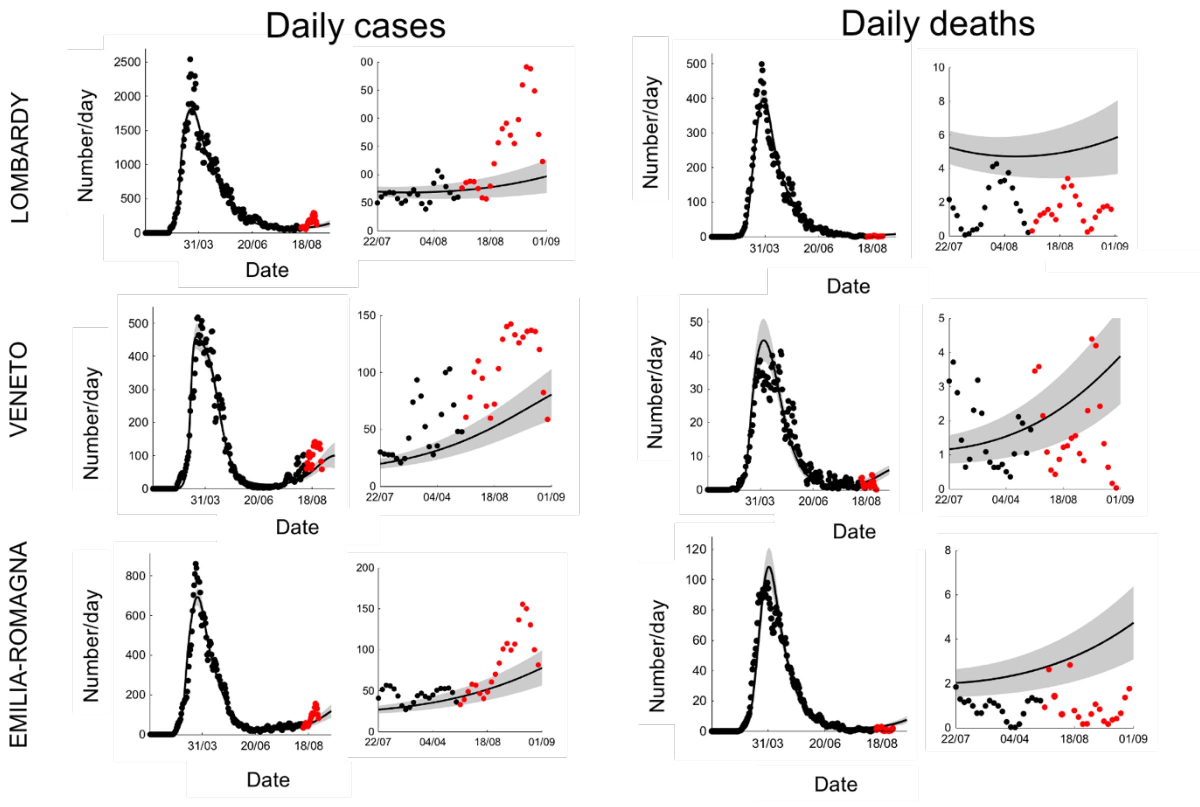

Fig. SM-5. ***Model predictive validity on dataset 2 (Lombardy, Emilia-Romagna and Veneto).*** *Left*. Black dots represent daily data (7-day sliding-window average) of positive cases reported by the "National Civil Protection Agency" for the six regions under investigation. Black lines represent the posterior expectations following model inversion. Gray bands represent the 90% credible intervals. Red dots correspond to data of reported positive cases from August 11st to August 31th that were excluded from data fitting, in order to validate the model prediction on the time interval shown in the inset. *Right*. Similarly, plots show the reported daily deaths from January 22nd to August 10th (black dots) and the corresponding fitting with 90% credible intervals (black lines and gray bands). Red dots represent daily deaths in the subsequent 20 days along with model prediction and 90% credible interval (inset).

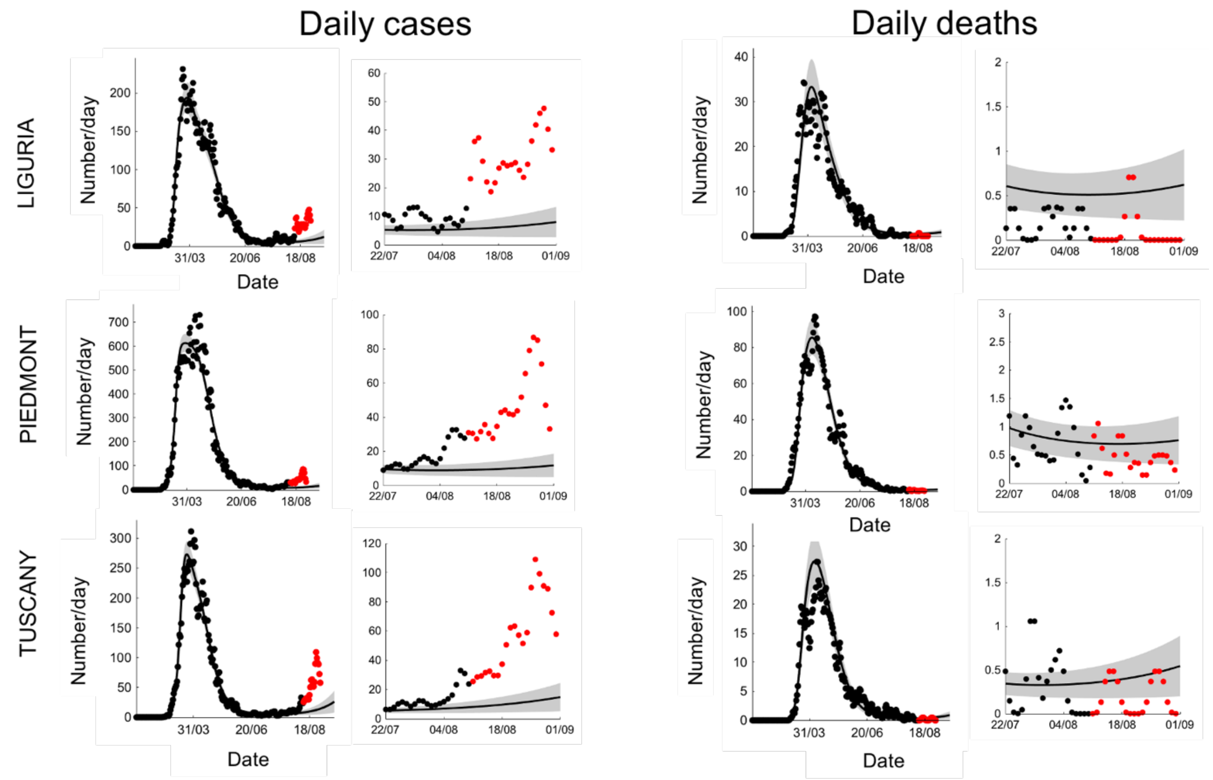

Fig. SM-6. ***Model Predictive validity (Liguria, Piedmont and Tuscany).*** This figure uses the same format as figure 2.

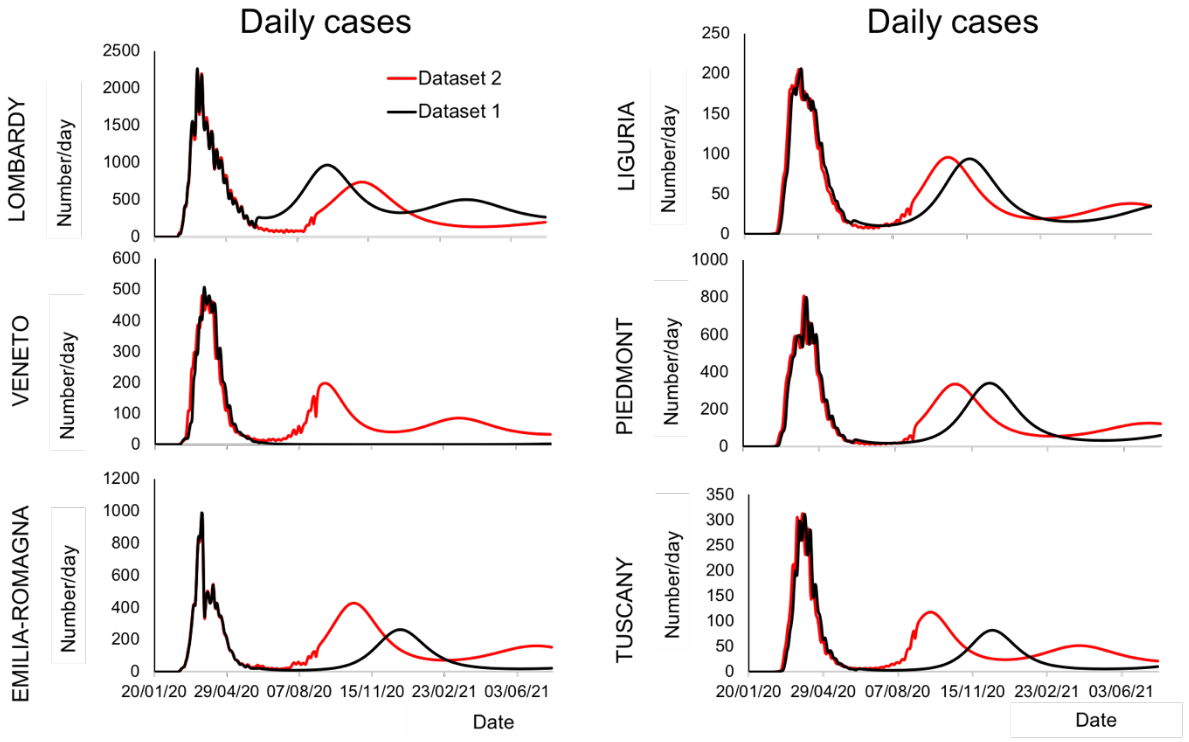

Fig. SM-7. ***Dataset 1 and Dataset 2.*** The panels show the comparison between prediction obtained by model inversion alternatively using dataset 1 (black lines) and dataset 2 (red lines) for the six regions under investigation. Note that in all cases but Veneto, the model predicted the occurrence of a second wave when dataset 1 was used as input.

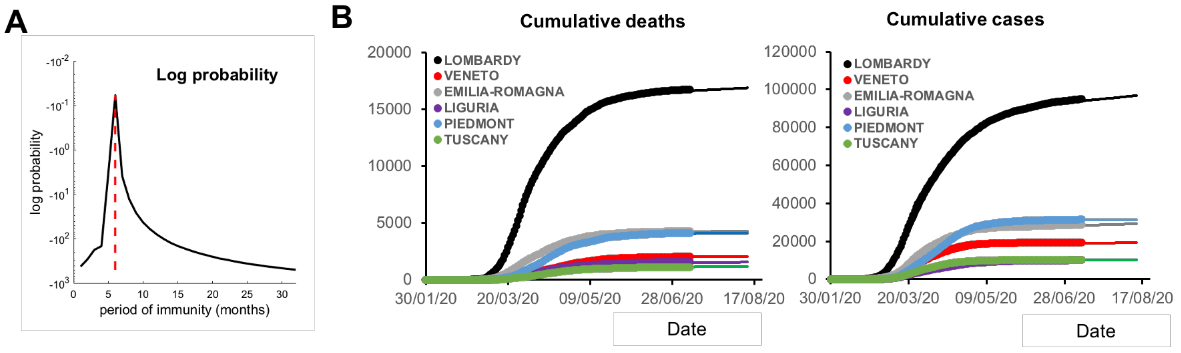

Fig. SM-8. ***Loss of protection from infection.*** **A** Posterior density (black line) is plotted on a log scale over the period of immunity based upon the marginal likelihood of models assuming a particular prior expectation (the prior covariance is 1/256). Red vertical dashed correspond to the peak of the curve (six months) **B.** The accuracy of model inversion is shown by plotting the empirical data for cumulative deaths (*left*) and cumulative new cases (*right*). The empirical data are shown as large dots whose time course overlaps that of the model prediction (thin lines).

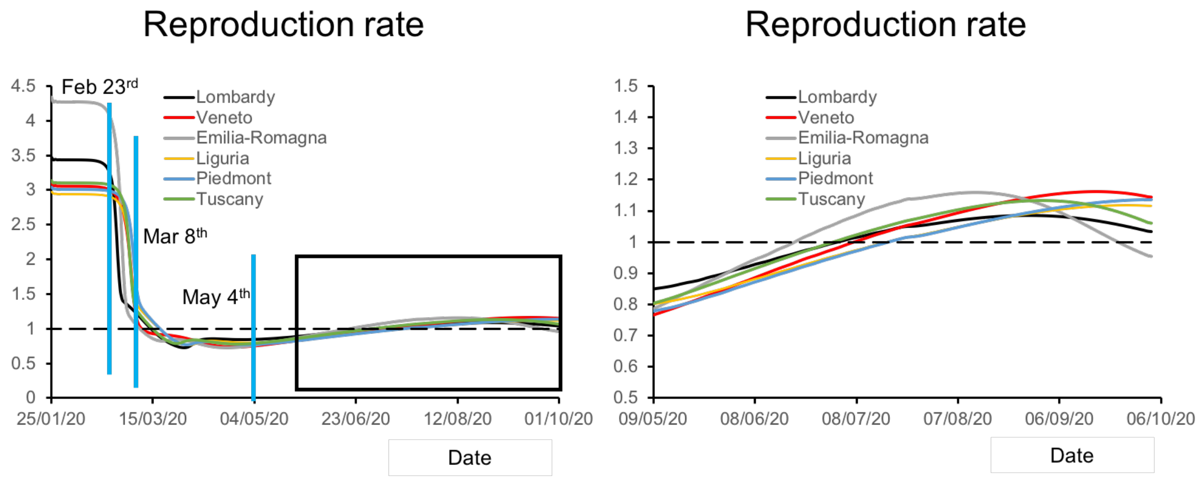

Fig. SM-9. Effective reproduction rate. Estimates of the effective reproduction rate, R_t for the six regions under consideration. *Left.* All regions (solid lines) show an initial R level above

1 at the beginning of the pandemic. The model-inferred value of R falls rapidly below 1 in response to the lockdown imposed by the Italian government (blue vertical lines), while also likely reflecting the acquisition of immunity. Around the beginning of July, R exhibits a slight increase and crosses again the threshold of R=1 in all regions. *Right*. The plot is a zoomed in version of the results around the time of threshold crossing (dashed line), showing that different regions cross the threshold at different times (then the R values remain above 1 for a variable period subtending the rise of the second waves). This result is confirmed by the official R_t reports of the "Italian Minister of Health" (https://www.epicentro.iss.it/coronavirus/aggiornamenti).

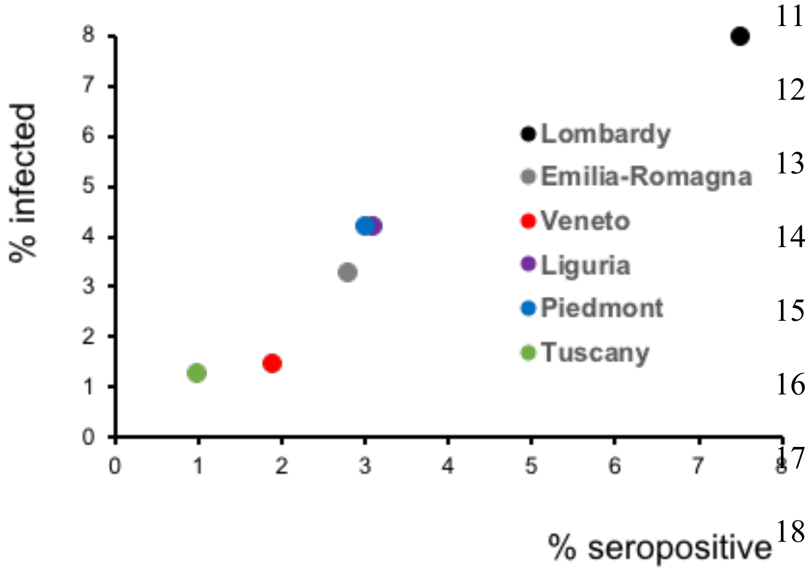

Fig. SM-10. ***Seroprevalence.*** The plot shows data on serological test (% of population that have been infected) against the peak value shown in Fig 3B of the main text, corrected by a factor accounting for the percent of positive cases and the total census population. Tuscany which had the lowest proportion was corrected the most, while Lombardy, showing the higher proportion of positive cases was not corrected.

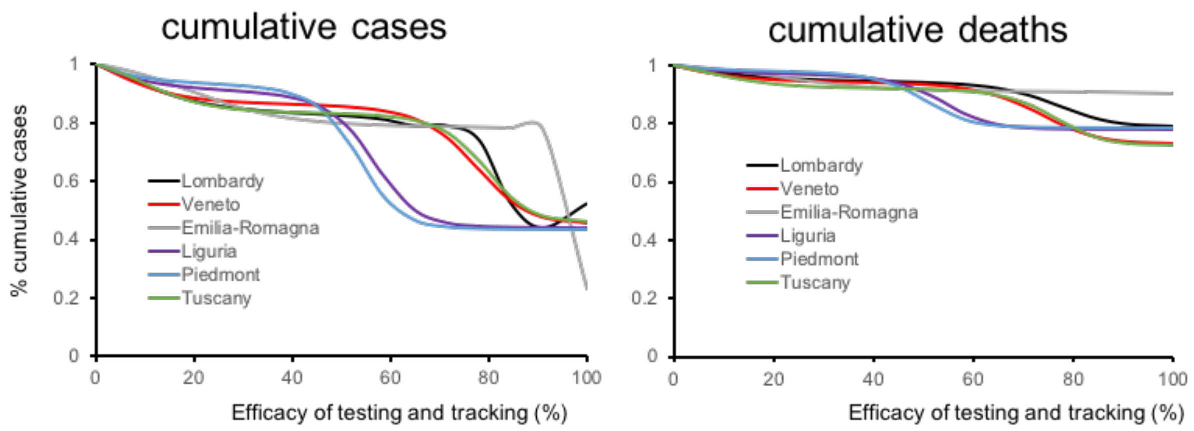

Fig. SM-11. ***Efficacy of testing and tracking.*** The plot shows the effects of increasing the efficacy of testing and tracking on the normalized cumulative cases (*left*) and on the normalized cumulative deaths (*right*). It should be noted that, as the efficacy increases, the number of cases and deaths tends to decrease, although with a variable impact on the different regions.

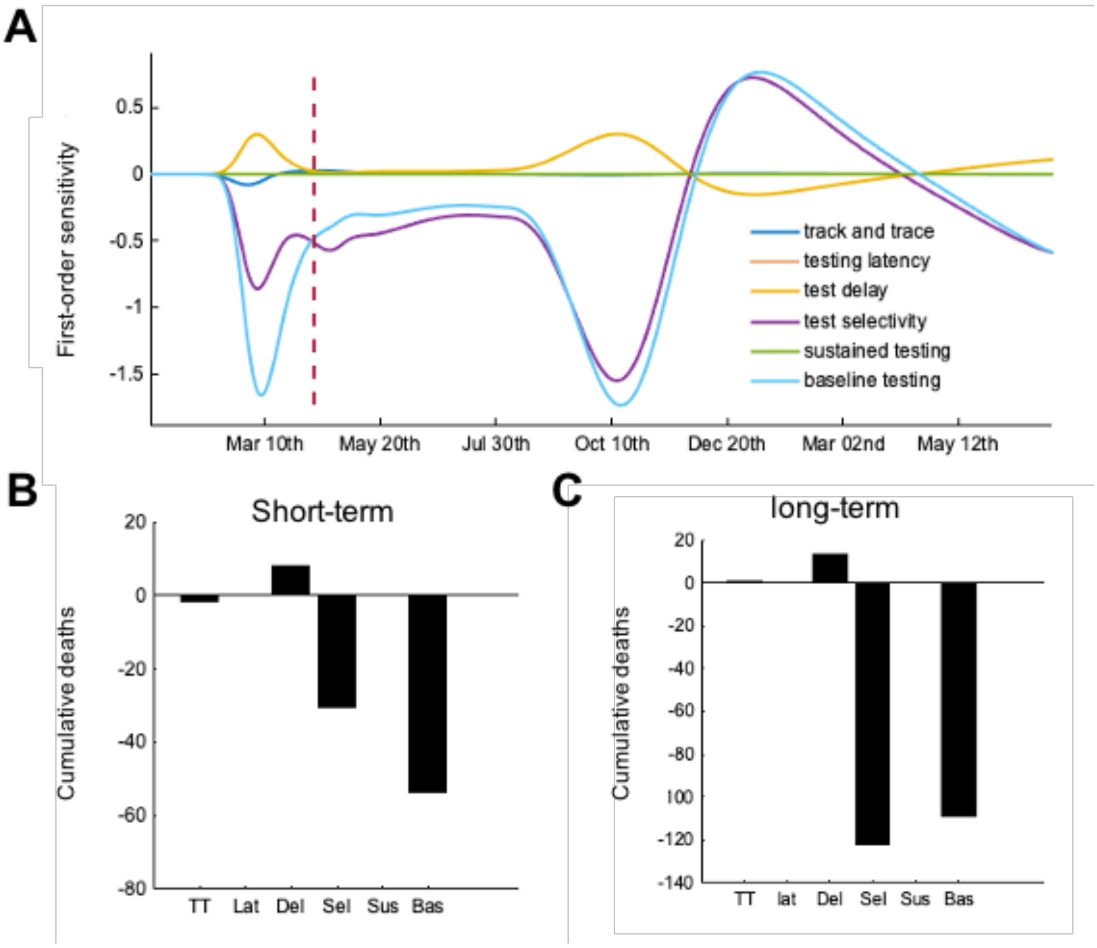

Fig. SM-12. ***Sensitivity analysis.*** **A.** The plot shows the change in death rates with respect to the logarithm of the parameters controlling the efficacy of tracing and tracking (blue, "TT"), testing latency (red line, "Lat"), test delays (yellow, "Del"), the selectivity for people who are infected (purple line, "Sel"), sustained testing (green, "Sus") and baseline testing (light blue, "Bas"). Positive values of first-order sensitivity mean that an increase in the parameter is associated with an increase in the outcome variable (new death cases), while negative values correspond to the opposite effect (an increase in the parameter is associated with a reduction in the outcome variable). The dashed vertical line separates the period of summing changes in cumulative deaths. **B**. Histogram sums changes shown in the upper plot over a period of 100 days prior to the peak of the first wave (from January 1st to April 10th). **C**. Histogram sums changes shown in the upper plot over a period subsequent to April 10th. Of note, increasing

testing selectivity (Sel) and baseline testing (Bas) tend to decrease death rate, while increasing test delays (Del) tend to increase death rate.

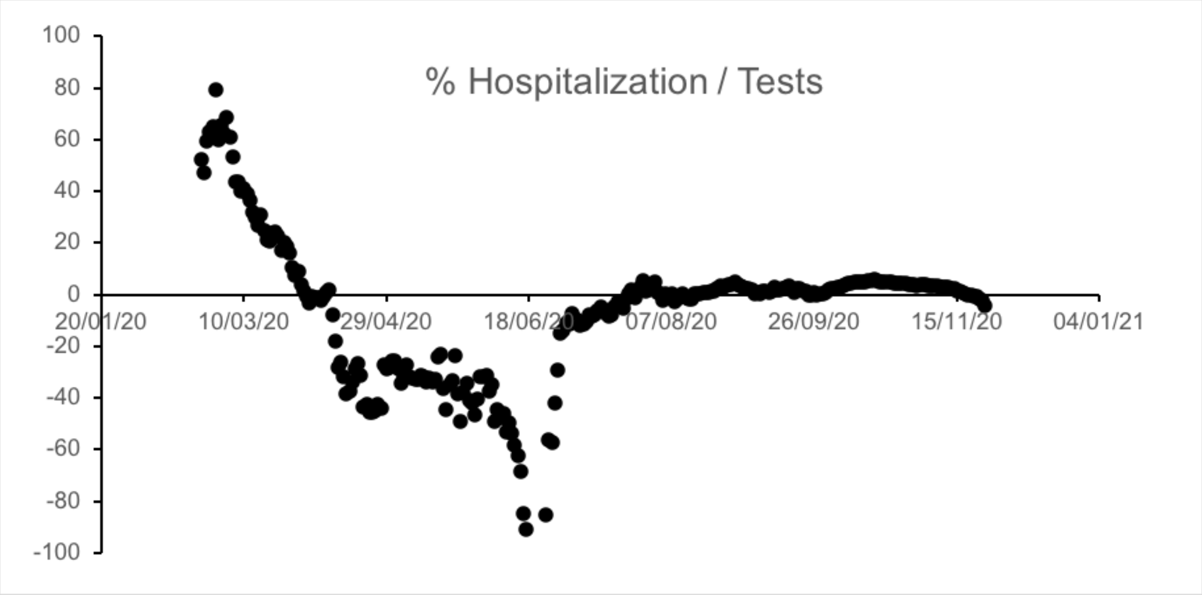

Fig SM-13 Hospitalization *vs* positive cases. The plot shows the temporal evolution of the proportion (%) of people that were daily hospitalized respect to the effective positive cases detected in Lombardy.

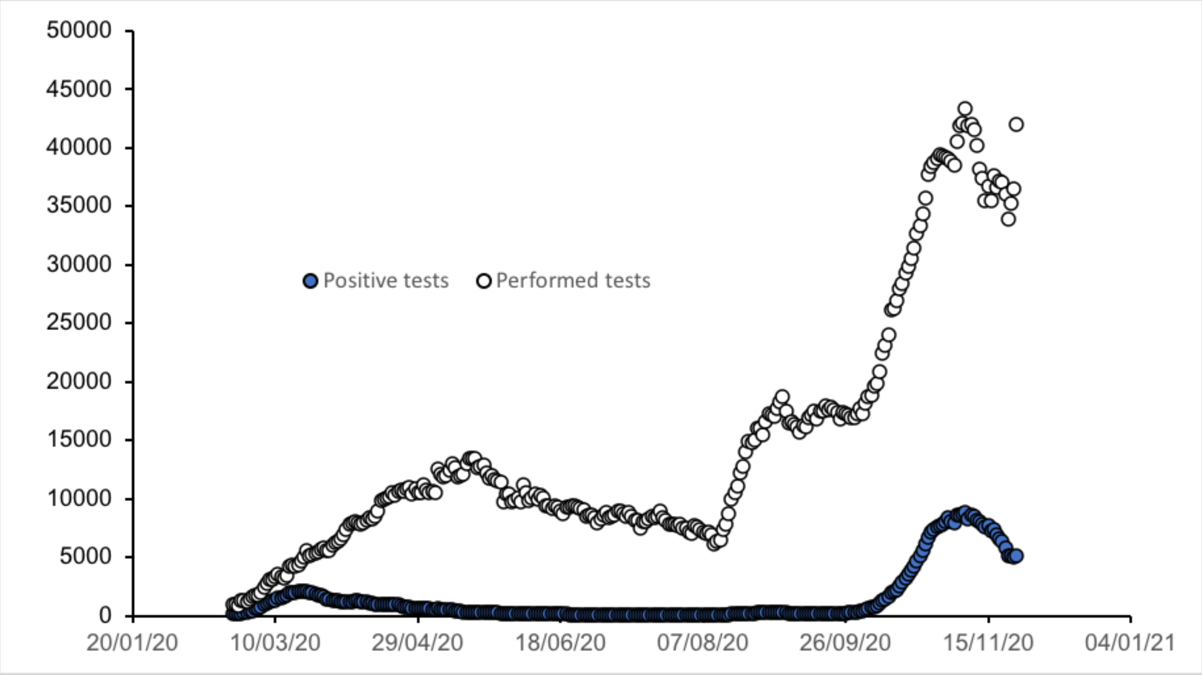

Fig SM-14 Positive cases and performed tests. The plot shows a comparison of the temporal evolution of the amount of positive cases effectively measured during the pandemic and the total number of daily tests performed in Lombardy.

PRIORS (TESTING)

a

Number	Parameter	Mean	Variance
1	Number of initial cases	4	1
2	Proportion of resistant cases	1/3	1/256
3	Population size (milions)	10 Lombardia; 4.5 Emilia-Romagna; 4.9 Veneto; 4.3 Piemonte; 1.3 Liguria; 3.7 Toscana	0
Location			
4	Probability of going out	1/3	1/256
5	Social distancing threshold	1/32	1/256
6	CCU capacity threshold (per capita)	16/100000	1/16
Infection			
7	Effective number of contacts:home	4	1/256
8	Effective number of contacts:work	48	1/256
9	Transmission strenght	1/3	1/256
10	Infected period (days)	4	1/16
11	Infectious period (days)	4	1/16
12	Period of immunity (months)	7	0
Symptoms			
13	Incubation period (days)	16	1/256
14	Probability of ARDS	1/128	1/256
15	Symptomatic period (days)	8	1/256
16	ARDS period (days)	10	1/256
17	ARDS fatality rate: CCU	1/3	1/256
18	ARDS fatality rate: home	1/8	1/256
Testing			
19	Efficacy of tracking and tracing	1/10000	1
20	Sensitivity of testing (months)	1/10000	1
21	Sustained testing	1/10000	1
22	Baseline testing	8/10000	1
23	Selectivity of testing infected people	1	1/16
24	Delay in reporting test results	2	1/256

PRIOR (BMC)

b

Number	Parameter	Mean	Variance
1	Number of initial cases	4	1
2	Proportion of non-susceptible cases	1/2	1/256
3	Effective population size (milions)	8	1
Location			
4	Probability of going out	1/3	1/256
5	Social distancing threshold	1/32	1/256
6	CCU capacity threshold (per capita)	16/100000	1/256
Infection			
7	Proportion of non-contagious cases	1/2	1/256
8	Effective number of contacts:home	4	1/16
9	Effective number of contacts:work	48	1/16
10	Transmission strenght	1/3	1/16
11	Infected period (days)	4	1/256
12	Infectious period (days)	4	1/256
13	Period of immunity (months)	from 1 to 32	1/512
Symptoms			
14	Incubation period (days)	16	1/256
15	Probability of ARDS	1/32	1/256
16	Symptomatic period (days)	8	1/256
17	ARDS period (days)	10	1/256
18	ARDS fatality rate: CCU	1/2	1/256
19	ARDS fatality rate: home	1/8	1/256
Testing			
20	Efficacy of tracking and tracing	1/10000	1
21	Latency of sustained testing (months)	2	1
22	Sustained testing	4/10000	1/256
23	Baseline testing	4/10000	1/256
24	Selectivity of testing infected people	8	1/16
25	Delay in reporting test results	2	1/256

Table SM-1: ***Parameter priors for Testing and BMC models.*** List of parameters, with corresponding prior values for mean and variance, used for inversion of Testing model (*left*) when input data were the number of daily serological tests, positive tests and deaths. When performing the Bayesian model comparison procedure (BMC model, *right*), input data were only the number of daily positive tests and deaths. Note that: i) priors n° 10,11,12,13,15,16 are time constants (τ), see section *"Model parameters and latent causes"* in the main text ii) variances are reported as log-values.

FACTORS	PARAMETERS	LOMBARDY	VENETO	EMILIA - ROMAGNA	LIGURIA	PIEDMONT	TUSCANY
Location parameters	Prob going out from home (%)	33.28	33.81	33.33	33.36	33.67	32.90
	Social distancing threshold	0.0275	0.0314	0.0336	0.0318	0.0329	0.0265
	Bed availability threshold (per capita)	0.000171	0.000164	0.000165	0.000165	0.000167	0.000166
Infection parameters	Effective number of contacts being at home	5.47	4.86	5.90	6.05	6.17	6.46
	Effective number of contacts being out	51.00	58.52	49.64	44.03	46.86	47.32
	Probability of getting contagion for each contact (%)	0.48	0.50	0.51	0.47	0.51	0.53
	Infected pre contagious period (days)	3.79	3.36	3.74	3.62	3.59	3.16
	Infected contagious period (days)	3.72	3.67	4.07	3.78	3.84	3.43
Clinical parameters	Time till symptoms (days)	17.53	17.91	18.14	17.70	18.49	20.97
	Prob severe symptoms from symptomatic conditions (%)	2.81	2.76	2.79	2.80	2.71	2.29
	Symptomatic period (days)	8.79	8.99	8.91	8.78	9.19	10.24
	CCU period (days)	14.90	15.10	14.62	12.73	16.77	13.40
	Prob of death from CCU with severe symptoms (%)	53.57	46.19	48.68	49.85	50.23	39.26
	Prob of survival from home with severe symptoms (%)	12.70	12.54	12.60	12.64	12.69	12.55
Testing parameters	Test, track and trace	0.0001	0.0001	0.0001	0.0001	0.0001	0.0001
	Test delays (days)	1.19	1.17	1.14	1.36	1.23	1.28
	Test selectivity (for infection)	11.20	4.32	9.00	9.17	10.85	4.11
	Baseline testing	0.059	0.107	0.075	0.05	0.03	0.029
Immunity	Proportion of resistant cases (%)	45.52	72.48	62.49	54.42	58.38	62.95
	Proportion of people with innate immunity (%)	48.33	59.78	49.05	48.78	50.62	50.72

Table SM-2. ***Posterior estimates of model parameters.*** Outcomes of the parameters estimates resulting from model inversion on dataset 2 including daily performed tests as input

Pearson's coeff	
LOMBARDY	0.87
VENETO	0.85
EMILIA-ROMAGNA	0.85
LIGURIA	0.94
PIEDMONT	0.97
TUSCANY	0.94

Table SM-3. ***Pearson's correlation coefficient.*** Results of the correlations between the inferred probability of leaving home and the effective mobility obtained from cellphone movements.

	RATIO BETWEEN 2ND AND 1ST PEAK		
	POSITIVE CASES (%)	SYMPTOMATIC (%)	INFECTED (%)
LOMBARDY	0.36	0.07	0.06
VENETO	0.40	0.14	0.11
EMILIA-ROMAGNA	0.40	0.11	0.09
LIGURIA	0.44	0.10	0.08
PIEDMONT	0.42	0.12	0.09
TUSCANY	0.29	0.11	0.09

Table SM-4. ***Ratio between the second and first peak.*** The values indicated in the table represent the ratio between the 1st and 2nd peak amplitude for the predicted time series of the

proportion of positive cases, symptomatic cases and infected individuals. Note that there is a significant reduction in the proportion of infected individuals, as well as in the proportions of symptomatic cases, compared to the overall positive cases.

FACTORS	PARAMETERS	LOMBARDY	VENETO	EMILIA - ROMAGNA	LIGURIA	PIEDMONT	TUSCANY
Population	Effective population	9.33	2.05	2.46	0.88	3.24	1.08
Location parameters	Prob going out from home	33.40	34.65	33.89	34.24	34.49	34.07
	Social distancing threshold	0.0307	0.0343	0.0338	0.0332	0.0357	0.0338
	Bed availability threshold (per capita)	0.000163	0.000167	0.000163	0.000160	0.000163	0.000165
Infection parameters	Effective number of contacts being at home	5.38	3.97	5.25	4.33	3.92	5.05
	Effective number of contacts being out	50.59	79.06	55.98	70.90	71.92	64.13
	Probability of getting contagion for each contact	47.46	54.65	51.13	53.36	49.04	56.27
	Infected pre contacious period (days)	3.61	3.52	3.73	3.71	3.59	3.55
	Infected contacious period (days)	3.63	3.83	3.98	3.88	3.86	3.84
Clinical parameters	Time till symptoms (days)	15.38	17.87	16.36	15.97	16.22	17.59
	Prob severe symptoms from symptomatic conditions	3.12	2.80	3.08	3.13	3.10	2.83
	Symptomatic period	7.70	8.71	7.98	7.95	8.00	8.59
	CCU period	7.47	11.67	9.92	9.81	10.30	10.76
	Prob of death from CCU with severe symptoms	49.71	49.03	53.58	50.56	51.23	48.68
	Prob of survival from home with severe symptoms	12.47	12.58	12.62	12.52	12.53	12.58
Testing parameters	Test, track and trace	0.0001	0.0001	0.0001	0.0001	0.0001	0.0001
	Test latency (days)	2.60	4.91	1.18	1.48	1.41	4.73
	Test delays (days)	2.06	1.97	2.02	2.01	2.01	1.97
	Test selectivity (for infection)	9.44	11.11	7.97	7.79	7.85	12.05
	Sustained testing	0.00	0.00	0.00	0.00	0.00	0.00
	Baseline testing	0.037	0.041	0.039	0.039	0.039	0.042
Immunity	Period of immunity (months)	6.37	5.72	5.93	6.30	6.04	6.02
	Proportion of resistant cases	50.09	65.44	55.02	52.33	61.46	58.66
	Proportion of people with innate immunity	52.80	47.84	49.14	52.98	51.48	50.57

Effective population	9.23	2.05	2.46	0.88	3.24	1.08
Population size (in millions)	10	4.9	4.5	1.3	4.3	3.7
Effective % population	92.26	41.80	54.63	67.70	75.30	29.26

Table SM-5. Posterior estimates of model parameters obtained following the procedure of Bayesian model comparison (i.e. period of immunity set to 7 months).
